# Structural Changes in Isometrically Contracting Insect Flight Muscle Trapped following a Mechanical Perturbation

**DOI:** 10.1371/journal.pone.0039422

**Published:** 2012-06-25

**Authors:** Shenping Wu, Jun Liu, Mary C. Reedy, Robert J. Perz-Edwards, Richard T. Tregear, Hanspeter Winkler, Clara Franzini-Armstrong, Hiroyuki Sasaki, Carmen Lucaveche, Yale E. Goldman, Michael K. Reedy, Kenneth A. Taylor

**Affiliations:** 1 Institute of Molecular Biophysics, Florida State University, Tallahassee, Florida, United States of America; 2 Department of Cell Biology, Duke University Medical Center, Durham, North Carolina, United States of America; 3 Medical Research Council Laboratory of Molecular Biology, Cambridge, United Kingdom; 4 Pennsylvania Muscle Institute, University of Pennsylvania, Philadelphia, Pennsylvania, United States of America; 5 Institute of DNA Medicine, Jikei University School of Medicine, Tokyo, Japan; University of Minnesota, United States of America

## Abstract

The application of rapidly applied length steps to actively contracting muscle is a classic method for synchronizing the response of myosin cross-bridges so that the average response of the ensemble can be measured. Alternatively, electron tomography (ET) is a technique that can report the structure of the individual members of the ensemble. We probed the structure of active myosin motors (cross-bridges) by applying 0.5% changes in length (either a stretch or a release) within 2 ms to isometrically contracting insect flight muscle (IFM) fibers followed after 5–6 ms by rapid freezing against a liquid helium cooled copper mirror. ET of freeze-substituted fibers, embedded and thin-sectioned, provides 3-D cross-bridge images, sorted by multivariate data analysis into ∼40 classes, distinct in average structure, population size and lattice distribution. Individual actin subunits are resolved facilitating quasi-atomic modeling of each class average to determine its binding strength (weak or strong) to actin. ∼98% of strong-binding acto-myosin attachments present after a length perturbation are confined to “target zones” of only two actin subunits located exactly midway between successive troponin complexes along each long-pitch helical repeat of actin. Significant changes in the types, distribution and structure of actin-myosin attachments occurred in a manner consistent with the mechanical transients. Most dramatic is near disappearance, after either length perturbation, of a class of weak-binding cross-bridges, attached within the target zone, that are highly likely to be precursors of strong-binding cross-bridges. These weak-binding cross-bridges were originally observed in isometrically contracting IFM. Their disappearance following a quick stretch or release can be explained by a recent kinetic model for muscle contraction, as behaviour consistent with their identification as precursors of strong-binding cross-bridges. The results provide a detailed model for contraction in IFM that may be applicable to contraction in other types of muscle.

## Introduction

One of the oldest methods for studying the mechanical properties of active muscle is the application of a rapid mechanical perturbation to an isometrically contracting muscle fiber. In a steady state contraction, myosin cross-bridges generate force asynchronously, which makes it difficult to distinguish the structural changes associated with the individual steps of the cross-bridge cycle because most experimental techniques typically produce only an average measurement over the ensemble. The application of a sudden length perturbation (e.g. 0.12 ms) to an isometric contraction can force attached cross-bridges to respond simultaneously [1], [2].

**Figure 1:**
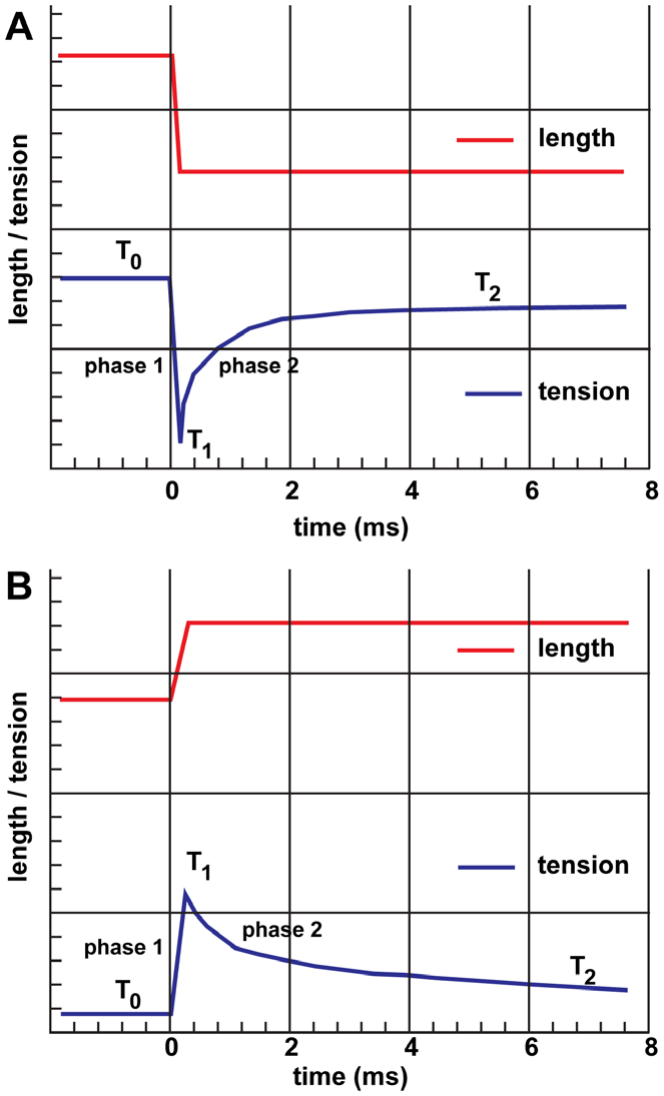
Typical mechanical traces. (A) Following a quick release of 4.5 nm/half sarcomere from isometric tension. (B) Following a quick stretch of 3 nm/half sarcomere from isometric tension. Adapted from reference [2].

When an isometrically contracting muscle at force T_0_ is allowed to shorten abruptly by a small amount and then held constant at the new, shorter length, tension decreases simultaneously to T_1_ due to the reduced load on elastic elements, which reside in the filaments and cross-bridges (Fig. 1A). This response of the cross-bridges is referred to as phase 1. Force then initially recovers rapidly to T_2_ (phase 2), which is attributed to the active rotation of lever arms of attached cross-bridges, followed by a slower force recovery or even a slight reversal (phase 3) and a final, slower asymptotic recovery to a new isometric force level T_4_ (phase 4) [2]. For very small step-releases of <5 nm/half sarcomere, the phase 2 recovery is complete during the first 2 ms, indicating that continuously attached cross-bridges undergo a power stroke to restore tension toward the isometric value [1], [3], [4]. Quick releases ≥12 nm/half sarcomere drop the tension so far that the phase 2 recovery is absent and tension redevelops slowly through phases 3 and 4.

Muscle fibers become stiffer during a stretch, suggesting that force enhancement by stretch is partly related to the recruitment of additional elastic components [5]. When an isometrically contracting muscle at tension T_0_ is suddenly step-stretched and held at the new length, the force increases during the stretch from T_0_ to T_1_ due to additional strain on the elastic elements, which consist of myosin heads and the filaments themselves (Fig. 1B). Part of this strain is born by the filaments, which elongate slightly and, it is thought, second heads of actin-attached myosin, which bind the thin filament [5]. Tension decreases from T1 to T2 during the next few milliseconds as the thick filaments return to their original length at the expense of the cross-bridges whose lever arms “back bend” further to accommodate the higher loading force imposed on them [5].

Myosin heads consist of a motor domain (MD), which has the catalytic and actin binding functions, and a lever arm that amplifies small structural changes within the MD to produce filament sliding. The lever arm consists of a small folded domain, dubbed the converter, a long α-helical peptide that binds the two light chains, an essential light chain, ELC, and a regulatory light chain, RLC. Attached fluorescent or paramagnetic probes can monitor separately the MD and the lever arm.

Fluorescence polarization measurements show that probes bound to the RLC of myosin in muscle fibers tilt in response to quick length changes during active contraction [6], [7], [8]. Similar measurements on contracting/active muscle fibers labeled at Cys707 of the myosin MD show no change in probe orientation in response to length changes [9], suggesting that the myosin head contains an internal hinge, probably between the nucleotide-binding domain and the light chain region. Motions of the lever arm around this hinge rather than changes in MD orientation may underlie force generation. The detected orientations of probes attached to light chains for active isometric contraction showed a broad distribution of tilt angles. Based on stiffness information and filament compliance, it was estimated that the lever arms of 20% of the heads tilt in response to a length step during active contraction [8]. However, how much of this motion is passively linked to filament sliding and how much to active force generation still remains a question.

X-ray diffraction of frog muscle has been used to probe with sub-ms precision the mechanically induced changes in average cross-bridge conformation that occur in synchrony with the induced force transients. Because of spacing differences, reflections on either the thick and thin filament can be used to identify changes occurring separately in either the lever arm or the myosin MD. Intensity of the M3 meridional X-ray reflection at 14.5 nm axial spacing, I_M3_, arises from the arrangement of myosin heads on the thick filament [10] and is used to report changes in tilt of cross-bridges during a force transient. A decrease in I_M3_ indicates tilting of myosin heads away from 90° and more parallel to the filament axis, which spreads the projection of their cross-bridge mass more uniformly along the filament axis [11], [12]. Conversely, intensity increases in the 37–38 nm, 5.9 and 5.1 nm layer lines can detect ordering of the MD interaction with the actin subunits.

When an active muscle fiber is rapidly shortened, I_M3_ decreases with about the same time course as the phase 2 rapid force recovery, but no detectable change in intensity occurs during the ∼0.1 ms length step itself [12]. Thus, the myosin head movements underlying the intensity decrease appear associated only with the slightly delayed active force generation early in phase 2, not with the length step and its elastically coupled, prompt force changes [12]. In contrast, when a quick stretch is applied to active muscle fibers, part of the intensity decrease of the 14.5 nm reflection occurs during the stretch, which reports the distortion of myosin heads due to the instantaneous elasticity of muscle and a small and fast reversal of the working stroke [13]. On the other hand, an X-ray study on muscle fibers utilizing a rapid temperature-jump to induce a tension rise found that the largest effect was found on the actin based layerlines rather than I_M3_ suggesting that a disordered to ordered transition of the MD on actin, rather than a simple lever arm tilt, was associated with the tension increase [14]. Evidence for this disorder-to-order transition of the MD early in force generation had also been provided by earlier EPR studies [15], [16].

X-ray diffraction has the advantage that the diffraction intensities can be recorded in real time from intact muscle fibers, but with the limitation that the measurements are an average of the ensemble of cross-bridges within the filament lattice. While shifts in this average can be detected, it is not straightforward to translate such shifts into changes of individual cross-bridges or the range and distribution of changes across the ensemble [17]. X-ray techniques are sensitive to the mass of the entire head, and do not resolve which fragments of the head bend or tilt. For example, I_M3_ measures the ordered mass along the 14.5 nm axial periodicity, but this mass could be due to myosin heads ordered with respect to the actin subunits and thus generating force, or they could be disordered with respect to the actin subunits, as would be typical of weak myosin-thin filament interactions. Like X-ray diffraction, electron microscopy (EM) also measures the cross-bridge ensemble but with the distinct difference that individual cross-bridges can be visualized at the same time.

Insect flight muscle (IFM) possesses a highly ordered paracrystalline arrangement of myosin and actin filaments that provides detailed X-ray diffraction patterns [18] and electron micrographs [19], [20]. Its filament arrangement is ideal for EM because the placement of an actin filament exactly midway between myosin filament pairs permits visualization of all the cross-bridges that attach to it within a section 25–30 nm thick. IFM also permits comparison of two activation pathways, one mechanically triggered by stretching partly activated muscle at pCa <6.0, referred to as stretch activation, the other an isometric contraction induced by saturating calcium at pCa <4.5, that is mechanically equivalent to the same state in vertebrate striated muscle.

*Lethocerus* IFM exhibits force transients, similar to those of vertebrate skeletal muscles [1], [21]. Step changes in sarcomere length of 100 µs duration can be imposed on fibers in Ca^2+^-activating solution at the plateau (T_0_) of isometric tension. The quick recovery rate increases in the isometric contraction transients, going from the largest stretch to the largest release, indicating that the cross-bridge kinetics of *Lethocerus* IFM have a strain dependence similar to that in skeletal fibers from vertebrate muscle.

The fully Ca^2+^-activated isometric contractions in IFM have been developed as an experimental model, called High Static Tension (HST), to indicate maximum active force with no stretch activation [22], [23]. Our recent EM study of isometric HST (iso-HST) utilized improved data collection, dual axis electron tomography and focused classification of the cross-bridge distribution within the 38.7 nm structural repeats [24], [25]. This work resolved the actin subunits on the thin filament thereby enabling the fit of an actin filament atomic model to the density independent of the presence of strong-binding myosin heads, in turn enabling the separate identification of both strong- and weak-binding attachments. Strongly bound cross-bridges were found only in the region exactly midway between successive troponin (Tn) complexes. This region is defined as the target zone [26]. Two types of weak-binding attachments were found in and near the target zone, one set attached to actin, the other set contacting tropomyosin (TM). Yet another set of apparently weak attachments were observed contacting Tn. Quasiatomic models of strongly bound attachments show a 77° sweep of lever arms spanning a 12–13 nm power stroke. A plausible sequence of weak-binding attachments toward the strong-binding configuration suggested that the weak-to-strong transition involved primarily azimuthal movements of myosin which may explain temperature jump experiments on isometrically contracting muscle [14] as well as providing a mechanism for myosin heads to cycle in place during active contraction [23].

Here we report on further investigations into the HST state of IFM using a 2 ms duration stretch (str-HST) or release (rls-HST) and their effect on both the structure and distribution of cross-bridges as they adjust to the mechanical perturbation. Changes relative to iso-HST include some new structures and a significant change in the distribution and frequency of previously observed structures. An increase in the number of two-headed attachments occurred after a quick stretch. Following a quick release the number of mask motifs in which myosin heads from successive 14.5 nm levels (crowns) contact a single target zone, as well as myosin heads contacting Tn (troponin bridges) increased. After a mechanical transient, there was also a dramatic change in the number and distribution of weak attachments in or near the target zone. Changes to the cross-bridge lever arm were comparatively small but consistent with the axial direction of the imposed length step.

## Results

### Tension Transients

**Figure 2:**
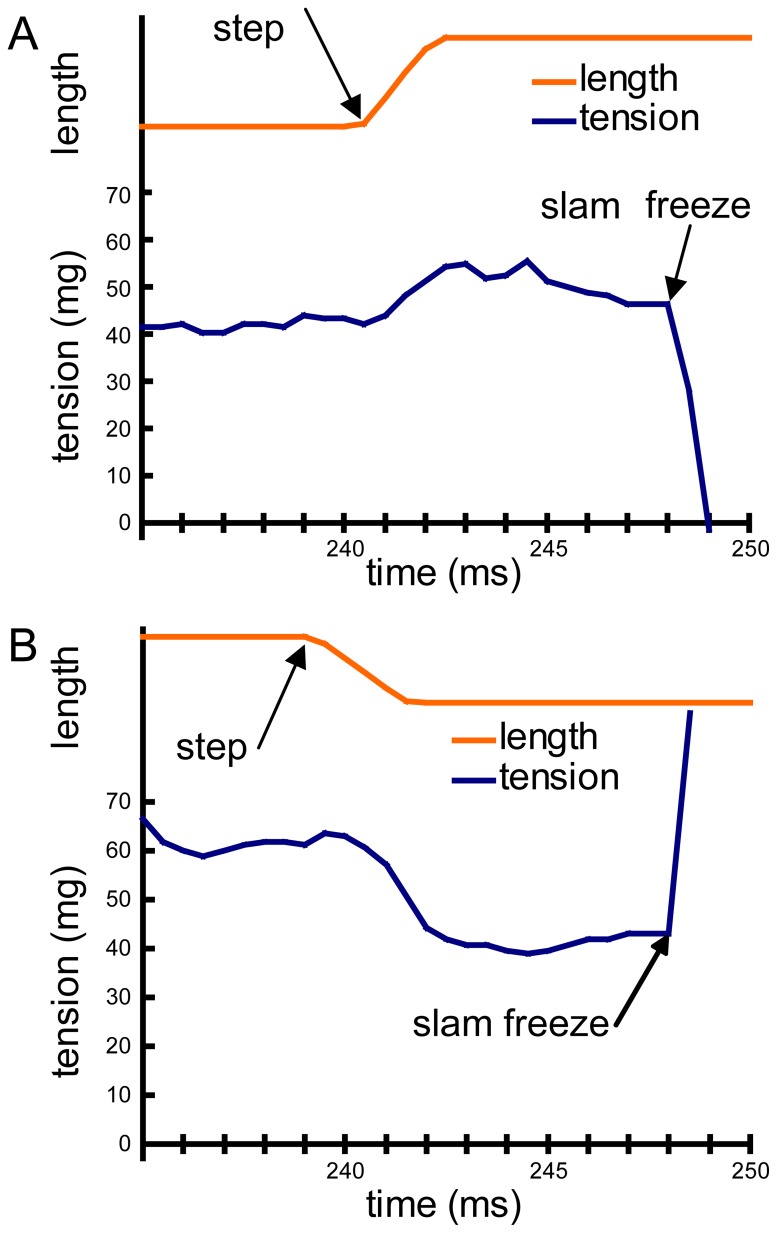
Experimental mechanical traces. (A) str-HST. (B) rls-HST. In both cases, the tension scale unit is mg. Scale for the length plot is arbitrary. See Table 1 for values in other units and for the actual length change.

The experiments reported here were severely constrained by the combined requirements for low-noise tension records of 10-µN resolution from single fibers slam-frozen with 1-ms precision and high-quality EM images of optimally oriented single-filament layers that were minimally perturbed by the slam-freezing impact. Although each experiment was repeated on at least 10 fibers, many with good mechanical traces failed to produce good quality EM specimens. The two specimens analyzed here were the best. In the step-stretch experiment (str-HST), the fiber was stretched 0.5% in 2 ms (Fig. 2A), and slam-frozen at the new length 5.5 ms later (Table 1). The force peaked at the end of the stretch, and had declined to near the pre-stretch level at the moment of freezing impact. In the step-release experiment (rls-HST), the fiber was released by 0.75% in 2.5 ms and the freezing impact occurred 6.5 ms later (Fig. 2B).

**Table 1:** Summary of fiber mechanics of iso-, str- and rls-HST.

	str-HST	rls-HST	iso-HST^1^
**Step length (%)**	0.50	0.75	–
**Step length (nm/half sarcomere)**	6	9	–
**Step time (ms)**	2.0	2.5	–
**Impact time (relative to start of** **step)**	7.5	9	–
**Tension before step (mg)**	44	62	–
**Peak tension after step (mg)**	57	39	–
**Tension at impact (mg)**	48	44	58.5
**Tension at impact (µN)**	470	431	573
**Target zone attachments/** **repeat** 2	1.89	2.157	3.012
**Strong binding attachments/** **repeat** 2	1.83^3^	2.022	2.134
**Force/target zone attachment** **(pN)** 4	3.10	2.49	2.37
**Force/strong binding attachment** **(pN)** 4	3.20	2.66	3.35

1 from reference [22].

2 A repeat represents a 38.7 nm length of the thin filament.

3 Includes out-of-target-zone attachments on actin subunit G.

4 Based on 5.7×10^8^ thick filaments/fiber cross section and 7.1 myosin heads per thin filament half repeat [22].

With the measured tension values and the known information on the filament arrangements in IFM (Table 1), the force generated by individual cross-bridges can be estimated by following previous calculations for iso-HST [22]. The force per strong-binding attachment varied from 2.66 pN for rls-HST to a maximum of 3.35 pN for iso-HST. For the three experiments, the mean is 3.07±0.36 pN, similar to values reported for vertebrate myosin [27], [28], [29], [30] although smaller forces have been reported for smooth muscle myosin II [31], [32].

### Microscopic Appearance of iso-, str-, and rls-HST

**Figure 3:**
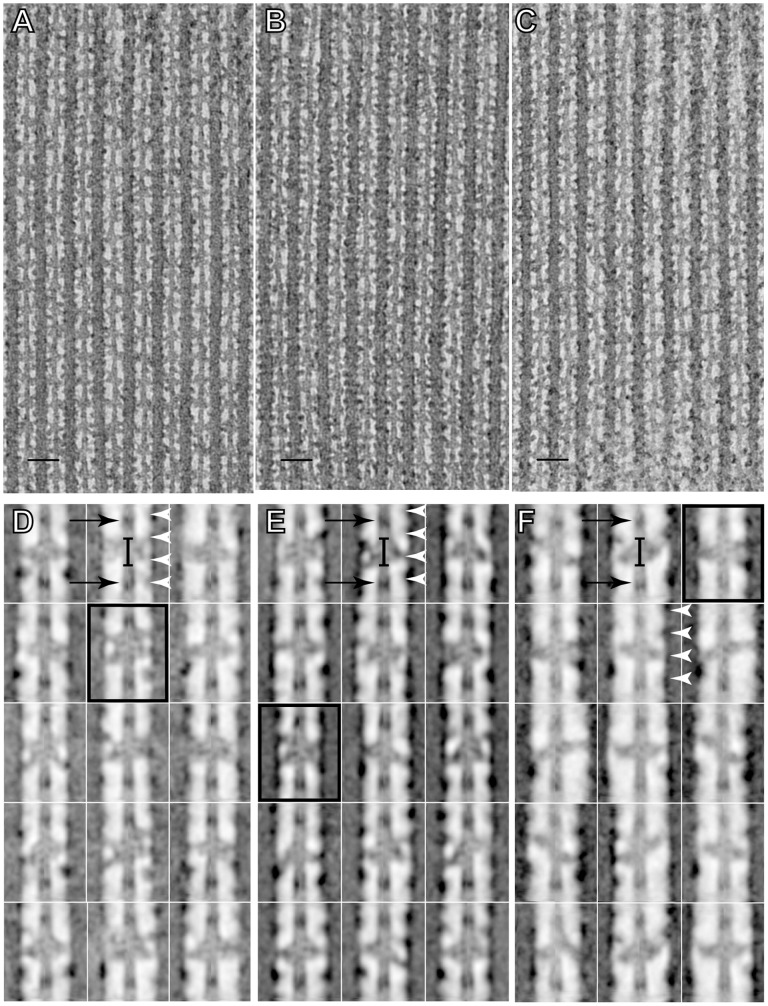
Projection images from each HST state. (A-C) Projection images of regions from each tomogram. (A) iso-HST state; (B) rls-HST;(C) str-HST. (D-F) Projection images from 15 reassembled primary mask class averages from each state. (D) iso-HST state; (E) rls-HST; (F) str-HST. In D-F, one mask motif structure has been outlined in each panel. Paired brackets show the location of the actin target zone; black arrows the Tn complex and white arrowheads the myosin head origins. Orientation has Z-line at the bottom, M-line at the top. Bar in the top panels is 50 nm.

The tomograms from all three HST states appear similar, with numerous cross-bridges bound to the thin filament (Fig. 3A–C). The 14.5-nm shelves of myosin heads on the thick filament are most visible in rls-HST, which had the lowest tension and the fewest attachments to actin, suggesting that the better-defined shelves are due to unattached heads that return to their original 14.5 -nm shelves.

Myosin head binding occurs all along the thin filament but is most frequent at the target zone, midway between the Tn complexes (Fig. 3D–F). A visual comparison of the class averages gives the impression that the cross-bridge lever arms in str-HST tilt upward toward the M-line (anti-rigor) more than those from rls-HST (Fig. 3E, F). However, the quantitative comparisons described below suggests that the average M-ward shift of the lever arm angle is quite small and the visual impression results mainly from certain stand alone, one-headed attachments, reinforced by a shift in the distribution of cross-bridge attachments along the actin filament toward the M-line for str-HST and toward the Z-line for rls-HST. Other differences are more striking, such as changes in the distribution of two-headed cross-bridges, weak-binding cross-bridges, and mask motifs.

### Distribution of Myosin-Actin Attachments

In an active contraction, we expect to find both weak and strong attachments to actin. As before [24], [25], we defined strong-binding attachments as those in which the myosin atomic model can be fit to the density without moving the MD away from the rigor acto-S1 structure [33], whereas weak-binding attachments are those that required moving the MD. We further divided weak attachments in or near the target zone depending on whether the MD contacted actin or tropomyosin (TM). Type 1 attachments contact actin and were only found in the target zone. Type 1 weak-binding attachments had their MD positioned on actin in a manner suggestive of a pre-power stroke stage. Hereafter, we will refer to these weak attachments as “pre-stroke” bridges acknowledging that this is a putative assignment. Type 2 attachments contact TM rather than actin and were found in and outside the target zone, but always on the M-line side. Hereafter we will refer to these weak attachments a “TM-bridges”, leaving open to question the mechanism by which they are maintained in contact with the thin filament.

**Figure 4:**
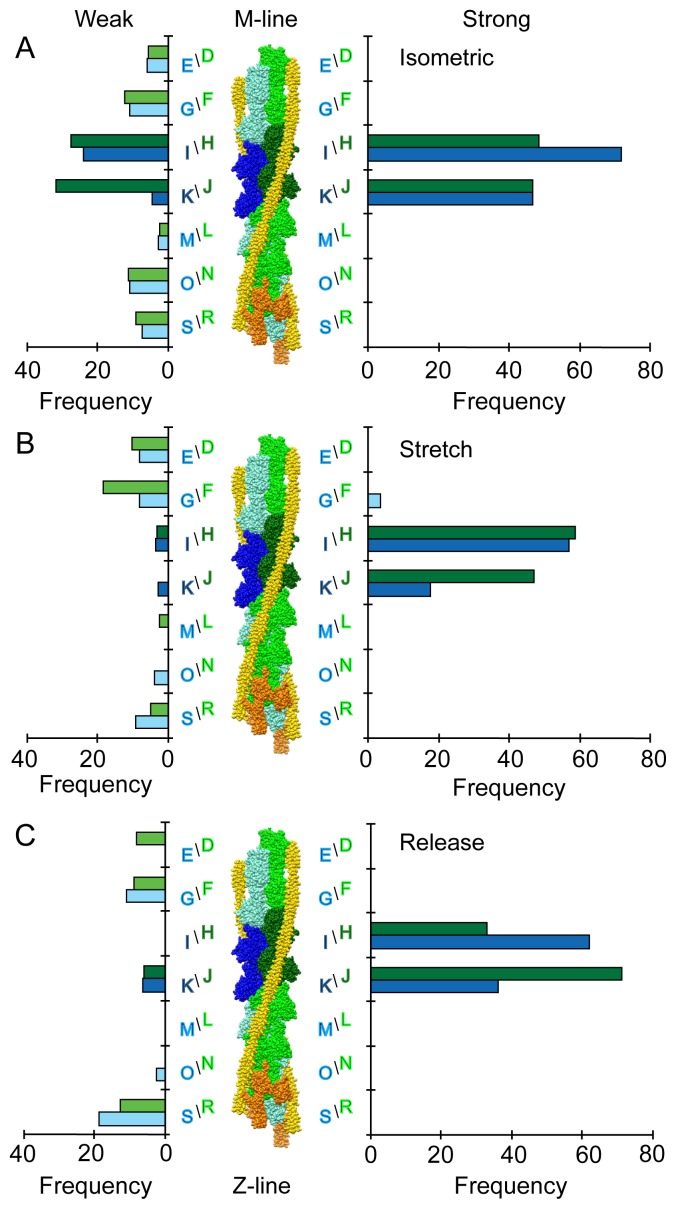
Distribution of cross-bridges for each actin subunit in the 38.7 nm axial period. The actin subunit designations are the same as those used for iso-HST [25]. Occupancy is given as a frequency, which means the total number of myosin heads, both weak and strong-binding, is divided by the total number of repeats in the data set for each state. On the right side are the occupancies for strong-binding attachments; on the left side are the occupancies for weak-binding attachments. Actin subunits H-K are target zone subunits; actin subunits R and S are bound to the Tn head complex. Orientation has Z-line at the bottom, M-line at the top. (A) iso-HST; (B) str-HST; (C) rls-HST.

Stretch or release did not significantly affect the number of strong-binding cross-bridges compared to iso-HST (Table 1), although the slightly smaller number of strong attachments in str- and rls-HST is consistent with the lower tensions in these states at the moment of freezing. In all three states, almost all strong-binding attachments are found on actin subunits H-K (Fig. 4, *right*), which confirms our prior identification of these subunits as the target zone [25]. The only violation of this restriction was a single strong-binding class average in str-HST found on actin subunit G just M-ward of the target zone representing only 1.8% of the total strong-binding attachments. Among the four target zone subunits, there are slightly more strong attachments on the M-ward subunits H/I in iso- and str-HST (Fig. 4A & B), whereas there are more on the Z-ward subunits J/K in rls-HST (Fig. 4C). Thus, stretch shifts the distribution of strong binding cross-bridges slightly M-ward, whereas release shifts it slightly Z-ward.

In contrast to the subtle effects on strong binding, stretch or release dramatically reduced the number and altered the distribution of weak-binding cross-bridges, especially within the target zone as can be seen by comparing the occupancy of weak-binding attachments at the target zone subunits H-K in iso-HST (Fig. 4A, *left*) to the occupancy in str- and rls-HST (Fig. 4B & C, *left*). In iso-HST, 29% of all target-zone attachments are weak-binding [25], but only 5% and 6% of the target zone attachments are weak binding in str- and rls-HST. Outside the target zone, stretch or release varied the distribution of weak-binding attachments less dramatically. In both states, the number of cross-bridges on actin subunits D/E, F/G, and L/M were roughly similar to those seen in iso-HST. Z-ward of the target zone, stretch or release reduced the number of cross-bridges at actin subunits N/O. Near the Tn complex at subunits R/S, stretch reduced the number of cross-bridges whereas release increased the number of cross-bridges relative to iso-HST.

Pre-stroke and TM-bridges responded differently to stretch or release (Table 2). In iso-HST, 72% of the weak-binding attachments within the target zone are pre-stroke cross-bridges, whereas the remaining 28% are TM-bridges. Most TM-bridges are found outside of the target zone, on the M-ward side [25]. Although a step release decreased the total number of weak attachments, it did not change the relative proportion of pre-stroke versus TM-bridges (71% : 29%). In contrast, a step stretch appears to have selectively reduced the number of pre-stroke bridges (14%), leaving TM-bridges (86%) to make up the majority of weak-binding attachments in str-HST. TM-bridges in str-HST were also more broadly distributed, being found at subunits E-I, in contrast to subunits F-H in iso-HST and only subunit G in rls-HST (Table 2).

**Table 2:** Summary of weak attachments for iso-, str- & rls-HST^1^.

State	Map ID	# found	Subunit^2^	Type^3^	Displacement^4^
**Isometric** 5		63	F	2	5.0–6.7 nm
		57	G	2	4.2–4.4 nm
		43, 99	H	2, 1	0.7–4.5 nm
		123	I	1	0.2–2.6 nm
		164	J	1	0.6–2.5 nm
		23	K	1	0.6 nm
**stretch**	25	26	E	2	8.2 nm
	129	23	E	2	4.0 nm
	144	28	F	2	4.2 nm
	44	41	G	2	2.6 nm
	348	36	H	2	3.2 nm
	144	43	I	2	2.5 nm
	104	31	K	1	7.1 nm
**release**	236	39	G	2	2.7 nm
	222	47	J	1	4.2 nm
	224	49	K	1	2.3 nm

1 These are obtained from the primary mask class averages.

2 Actin subunit as defined in Figure 4.

3 Type 1 are found in the target zone and are potential prepower stroke cross-bridges. Type 2 have their MDs contacting TM rather than actin.

4 Center of the MD from its center when in the strong binding position.

5 Summarized from Table 1 of reference [25].

### Reassembled Class Averages

**Figure 5:**
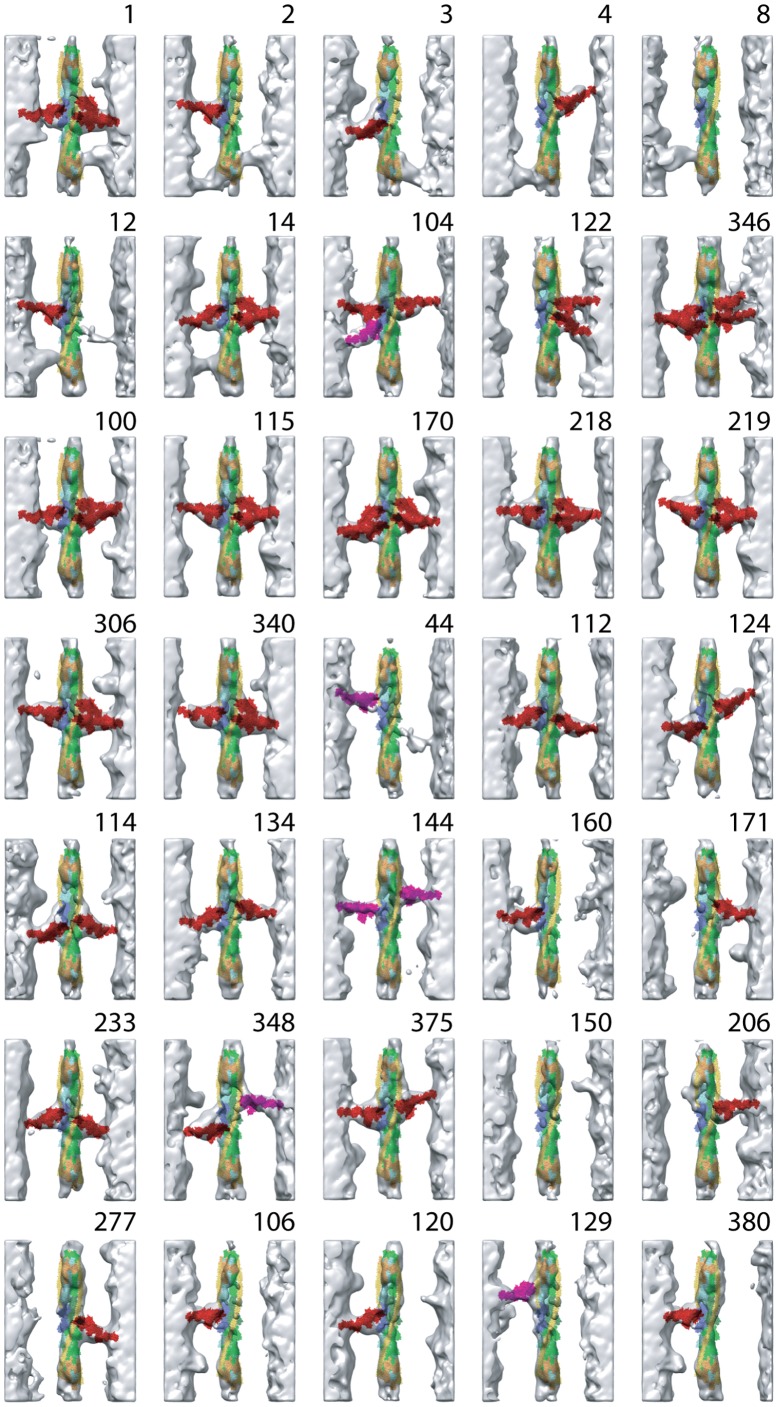
Reassembled averaged repeats for str-HST. Each reassembled repeat is a combination of one left-side and one right-side primary class and if necessary, between one and four troponin bridge class averages. Three repeats are included in Figure 7 and two, similar in appearance to 150 and having no myosin head attachments have been removed for considerations of space. These plus the three in Figure 7 include all the repeats to which quasiatomic models were built. The number in the upper right hand corner is the number assigned to the repeat. These numbers are referred to in the text and correspond to one of the raw repeats. Along the top two rows are those repeats with bridging density to the Tn complex. Quasiatomic models were not built for Tn bridges. Row 2 also contains all the mask motifs. Row 3 and the first two repeats in row 4 contain all the two-headed bridges. The rest are one-headed attachments of one form or another. The last two rows and the two repeats not included contain all the repeats that have no bridging density of any kind on one or more sides. Color scheme: strong-binding cross-bridges are red, weak-binding cross-bridges are magenta. Actin subunits are light green and light blue respectively with the target zone actins colored darker shades. Tn is colored orange, TM is colored yellow.

**Figure 6:**
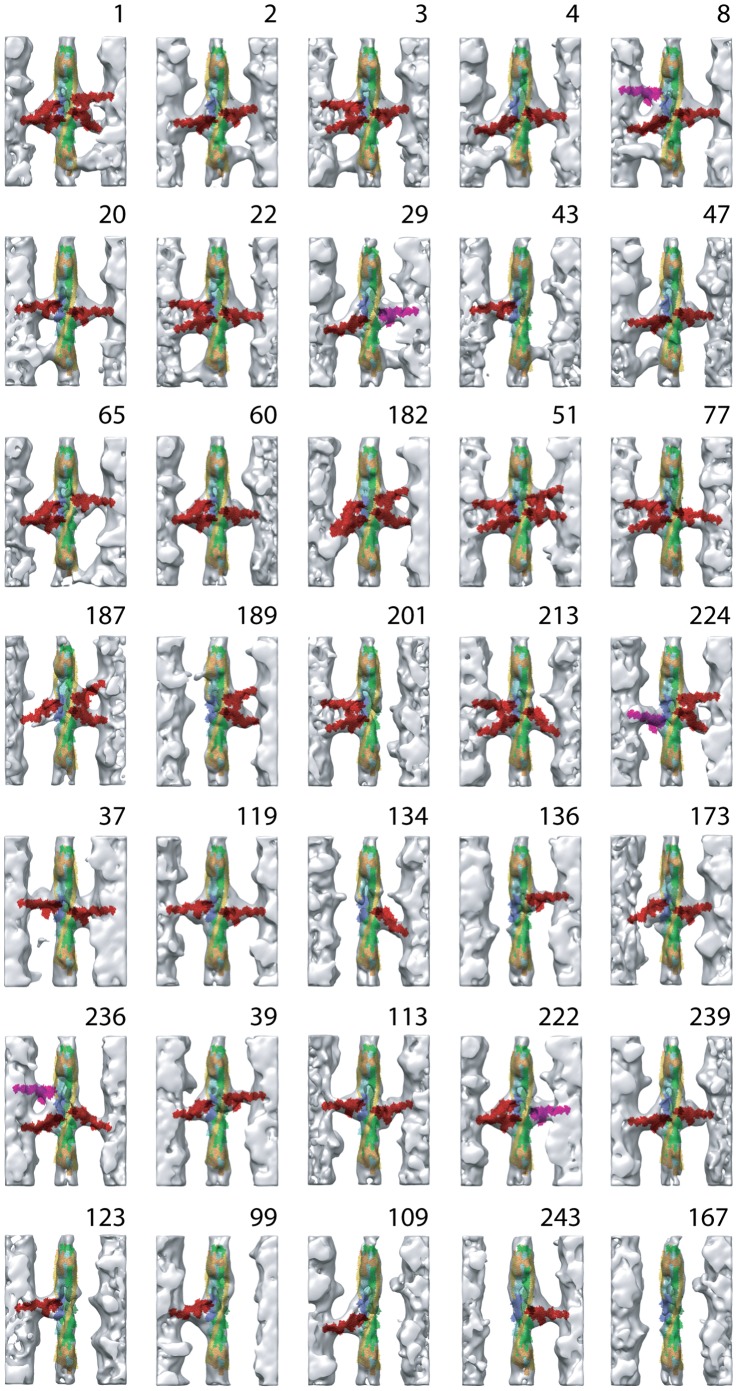
Reassembled averaged repeats for rls-HST. Same labeling and color scheme as for Figure 5. The top two rows contain Tn-bridge averages; row 3 contains two-headed bridge averages and mask motif structures; row 4 contains mask motif structures, while rows 5 and 6 are largely one-headed bridges of various types. The bottom row largely contains classes, which had no bridge marking on one or both sides.

**Figure 7:**
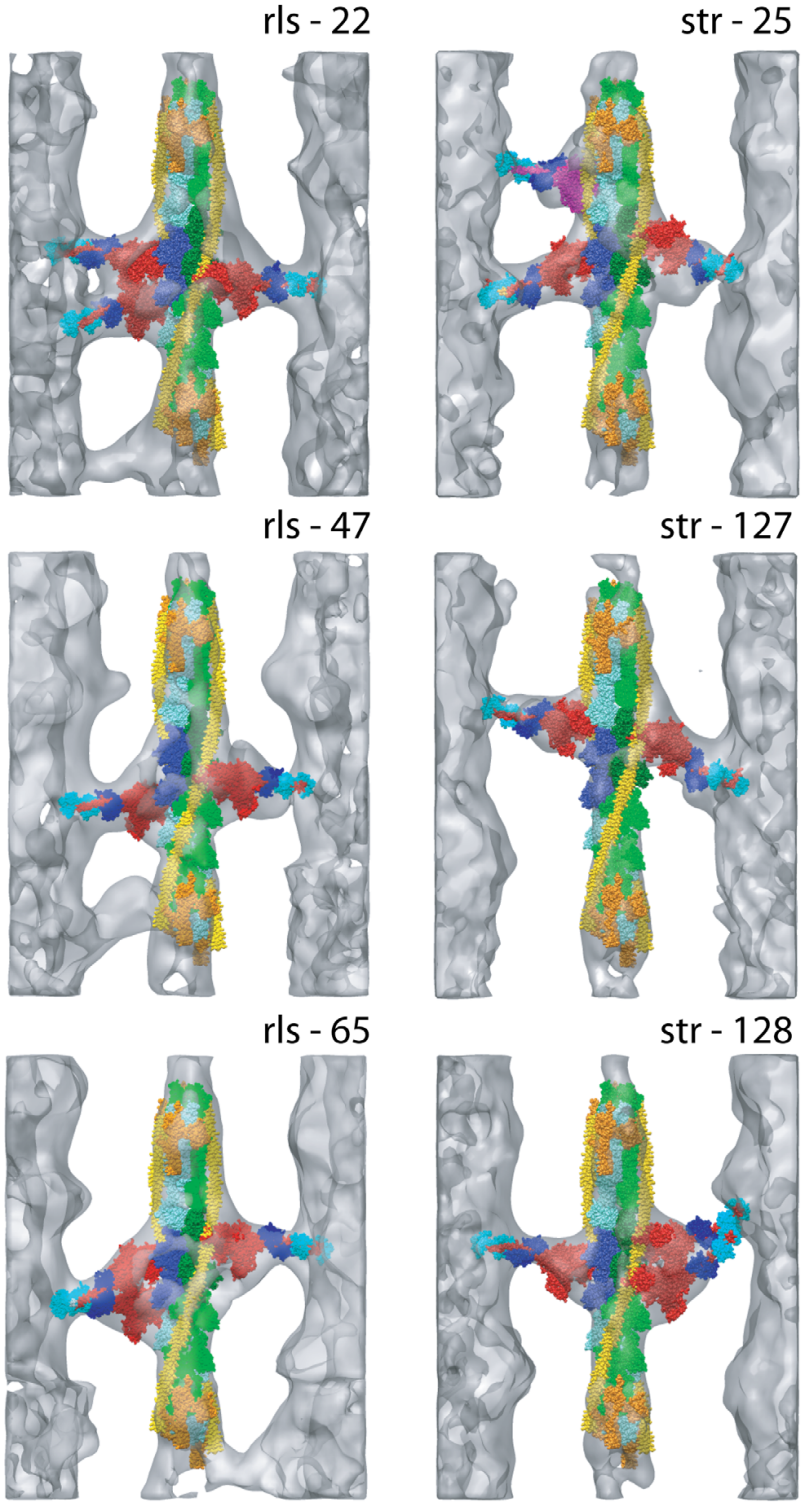
Quasiatomic models built for three str- and three rls-HST reassembled repeats. The top row contains mask motifs; the bottom row contains two-headed bridges. The three rls-HST models also have troponin bridge density, which is lacking in the str-HST models. Color scheme and labeling is same as for Figures 5 and 6 except that the ELC is colored dark blue and the RLC is colored cyan. Each of these is shown as Movies S1, S2, S3, S4, S5, S6.

Each raw repeat within the tomogram may contribute to several class averages, such as left- and right-side primary class averages, as well as troponin bridge classes. We reassembled the class averages to which an individual repeat contributed back into the original repeat, which improves the signal-to-noise ratio while retaining the rich variations in cross-bridge structure present in the tomograms [24]. The entire range of class averages can be represented by 40 reassembled repeats for str-HST (Figs. 5) and 35 reassembled repeats for rls-HST (Figs. 6). Three repeats each of str- and rls-HST including the single out-of-target zone strong binding class average (str-127) are shown at higher resolution in Figure 7.

Among the 40 reassembled repeats of str-HST (Figs. 5, 7), 13 were devoid of cross-bridges (e.g. repeats 8, 106, 120, 122, 150, 206, and 380). Eleven repeats contained two-headed cross-bridges (e.g. 1, 14, 100, 115, 128, 170, 218, 219, 306, 340, and 346). Six repeats contained troponin bridges (e.g. 1, 2, 3, 4, 8, 12, and 14). Four repeats contained mask motifs (e.g. 25, 104, 122, and 346). The rest contained one-headed, strong- or weak-binding myosin heads of various forms.

Among the 35 reassembled repeats of rls-HST, 8 lacked any cross-bridges (e.g. 99, 109, 123, 134, 167, and 243). Four repeats contained two-headed bridges (e.g. 1, 60, 65, and 182). Eleven repeats contained troponin bridges (e.g. 1, 2, 3, 4, 8, 20, 22, 29, 43, 47, and 65). Twelve repeats contained mask-motifs (e.g. 1, 3, 8, 22, 51, 77, 182, 187, 189, 201, 213, and 224). The rest contained one-headed, strong- or weak-binding cross-bridges of various forms. The individual cross-bridge forms are discussed below.

### Two-Headed Cross-bridges

Two-headed cross-bridges have both heads of a myosin molecule bound to adjacent actin subunits on one side of the actin filament (Table 3). In all three states, two-headed attachments were observed only in the target zone, never outside it. Three different varieties of two-headed bridges are possible based on the apparent binding strength of the MD to actin as defined above (strong-strong, strong-weak, or weak-weak pairs). All three were observed in iso-HST [25]. In contrast, only strong-strong two-headed attachments were observed in str- and rls-HST (Table 3). If all two-headed cross-bridges are compared without consideration of myosin head binding strength, the order is isometric>stretch>release. The order is stretch>isometric>release if only double strong attachments are considered. The lever arm axial angles of two-headed bridges show little difference between iso-HST and str-HST. rls-HST shows a large shift toward the rigor angle.

**Table 3:** Summary of 2-headed & mask motif structures.

	str-HST	iso-HST	rls-HST
**2-headed**			
^ 1^All	33.8	42.9	11.1
^ 2^Double strong	33.8	27.2	11.1
^ 3^M/Z angles	82°/108°	89°/114°	62°/88°
**Mask Motifs**			
^ 1^All	11.8	85.4	38.1
^ 4^Both on target zone	9.5	62.1	33.1

Values are calculated as: (number of structures)/(number of repeats) * 100. Because a single repeat might have two 2-headed bridges, the theoretical maximum is 200% (as seen in rigor). The same argument applies to mask motifs.

1 Includes all two-headed or mask motif attachments.

2 Means both heads are strong attachments.

3 The mean lever arm axial angle of heads bound to target zone actin subunits H and I on the M-ward side compared with that on actin subunits J and K on the Z-ward side.

4 Indicates both attachments occur on target zone actins H-K.

### Mask Motifs

We define the term “mask motif structure” to consist of two target zone or near target-zone attachments along a single actin strand, with the thick filament origins of the two attachments coming from successive 14.5 nm crowns. A complete mask motif would then consist of a pair of mask motif structures, such as rls-51 (Fig. 6), and resemble a harlequin mask. Mask motif structures are most frequent in iso-HST, less frequent in rls-HST, and least frequent in str-HST (Table 3). Most mask motif structures in str- (81%) and rls-HST (87%) have the two heads strongly bound to the two adjacent actin subunits within the target zone, e.g. rls-22 (Fig. 7), with a single exception found in str-HST, e.g. str-25 (Fig. 7). In contrast, 27% of the mask motifs in iso-HST featured two heads that were separated by an unoccupied actin subunit so that the Z-ward member was strongly bound within the target zone and the M-ward member weakly bound outside the target zone.

### Weak Actin-Myosin Attachments

**Figure 8:**
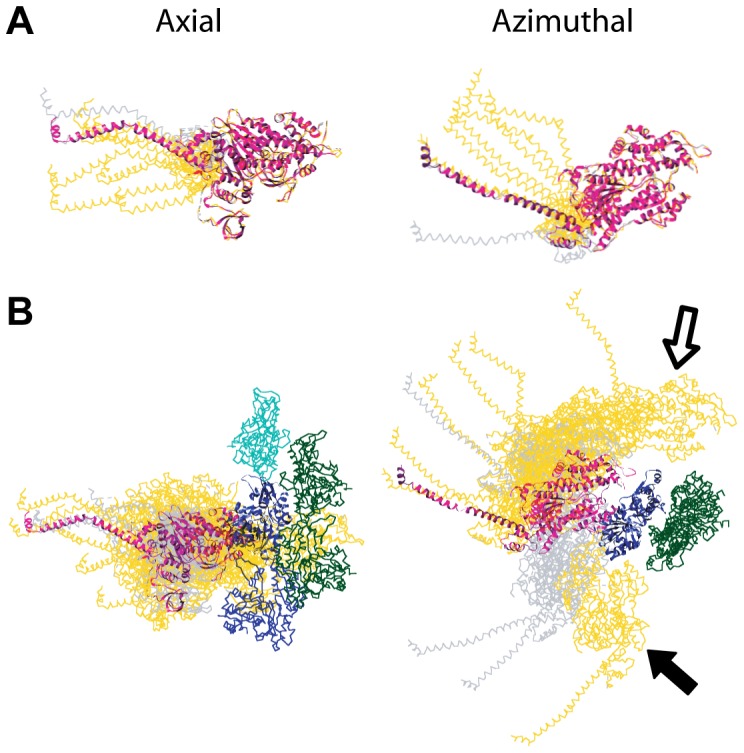
Composite views of weak-binding cross-bridge models. Axial views are oriented with Z-band at the bottom; azimuthal views are looking down the filament towards the Z-band. All weak-binding models were built starting from the scallop transition state structure (magenta) docked in the strong-binding configuration. In all panels, gold  =  str-HST, gray  =  rls-HST. (A) Weak-binding cross-bridge models superimposed onto the scallop MD. (B) Weak-binding models superimposed onto actin subunit I. The largest azimuthal MD displacements for pre-stroke and TM-bridges (filled and open arrows, respectively) were both found in str-HST.

The range of structural variation in weak-binding attachments can be appreciated by superimposing the quasiatomic models onto a common origin. With the MDs superimposed onto that of the scallop transition state structure (Fig. 8A), the image can be interpreted as inherent flexibility of the myosin head. The axial and azimuthal ranges after the length perturbation follow the same trends seen in iso-HST [25]. Axially, most weak attachments have the same lever-arm angle as the scallop transition state, and are thus not seen in the figure. Those that differ are distributed on either side of the scallop structure. Azimuthally in all three states, pre-stroke atomic models are anticlockwise with respect to the scallop structure and TM-bridge models are clockwise. Because most of the weak-binding bridges in str-HST are TM-bridges, their lever arm positions (Fig. 8A, *gold*) appear azimuthally clockwise with respect to the scallop structure. In contrast, more of the weak-binding bridges in rls-HST are pre-stroke bridges (Fig. 8A, *gray*) and therefore their lever arm angles appear anticlockwise with respect to the scallop structure.

Alternatively, the weak-binding quasi-atomic models can be aligned onto to a single actin subunit (Fig. 8B), in which case the variability of actin attachment is revealed. With this alignment, the models demonstrate the dominance of TM bridge attachments, which are seen as axially dispersed about the scallop structure. When viewed down the filament axis, the models describe a wide azimuthal arc about the central actin subunit. Pre-stroke attachments would need to move azimuthally clockwise around the actin subunit to convert to the strong-binding position, as previously described [25]. Although there may be some bias from the way the atomic models were constructed, we note that the surface of the lower 50 kDa domain of myosin nearly always faces the thin filament for all the HST weak binding bridges in and near the target zone. The upper 50 kDa domain rarely forms the contact site with actin.

### Axial Distribution of Lever Arm Angles

**Figure 9:**
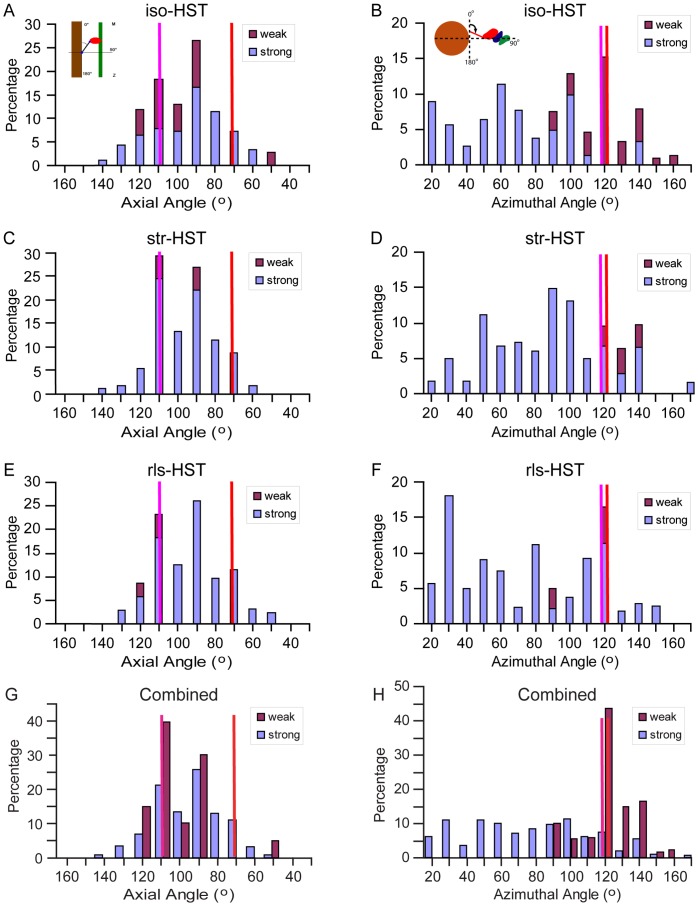
Angular ranges of lever arm angles for target zone bridges. iso-, str- and rls-HST are shown separately as well as the combined data from all three states. Panels A, C, E and G show axial lever arm angles, computed from the projection of the lever arm axis onto the fiber axis. Panels B, D, F and H show azimuthal lever arm angles after all primary mask class averages are transformed to thin filament actin subunit I. Sketches in the upper left hand of A and B show the angle convention. Vertical lines represent the initial structures used for the model building, red for rigor acto-S1 and magenta for the scallop transition state docked onto actin in the strong-binding configuration. Percentage value is calculated as: (number of attachments of this type in this range)/(total number of attachments of this type in this state) * 100. In this convention, the axial lever arm angle of the Holmes S1 structure is 70.5° and of the scallop transition state structure, 107°.

Previous X-ray diffraction studies of rapid stretches or releases in vertebrate muscles indicated that the lever arms of attached myosin heads tilt axially during both elastic and active force transients [5], [12], [34], [35], but because X-ray diffraction is indirect, its interpretation model-dependent, we sought to measure the indicated tilt by direct imaging of cross-bridges freeze-trapped late in the phase 2 response. We superimposed our quasiatomic models of all strong-binding attachments onto actin subunit I, and then superimposed all the MDs of the weak-binding attachments onto the MD of the strong-binding attachments. The weak-binding attachments might then be interpreted as reporting the intrinsic flexibility of myosin heads and the strong-binding attachments as reporting conformational changes required for force production *in situ* (Fig. 9).

When so aligned, the lever arms in all three states sweep out a broad axial range (∼80°) that is centered near to perpendicular (Fig. 9A, C, & E), consistent with previous studies [23]. Section compression could have enlarged the axial angular range by ±6°, as discussed previously [25]. Using the axial coordinate of residue 840 (Holmes structure) and 835 (scallop structure), the strong-binding lever arms sweep out an axial distance of 12 nm in str-HST and 13 nm in rls-HST. By comparison, the myosin crystal structures bracket an axial displacement between the same two residues of 6.2 nm. Weak-binding myosin heads within the target zone sweep out a smaller axial range, 27° (5 nm) for str-HST and 9° (2 nm) for rls-HST, than the strong-binding heads.

The distribution of strong-binding attachments appears bimodal, with peaks at 90° and 110°, which may be expected given that a cross-bridge binding to the target zone has a binary choice between two actin subunits that are about 20° apart axially. The different modes of the angular distributions, 90° in str-HST and 110° in rls-HST, are also consistent with the subunit distribution (Fig. 4, right), which shows more strong-binding attachments on M-ward subunits H/I in str-HST and more on Z-ward subunits J/K in rls-HST.

As expected, stretch shifted the mean axial tilt in the anti-rigor direction, and release in the rigor direction, but these changes were unexpectedly small and not statistically significant (Table 4). However, the extreme angles in the distribution also increased or decreased depending on whether the muscle fiber had been stretched or released. For example, the minimum of 50° (rigor-like) in rls-HST was not seen in str-HST, whereas the maximum of 140° (anti-rigor) in str-HST was not seen in rls-HST.

**Table 4:** Summary of axial lever arm tilt angles.

	str-HST	iso-HST	rls-HST
mean	97	96	93
min	63	54	54
max	135	147	135
st. dev.	18	23	21

**Figure 10:**
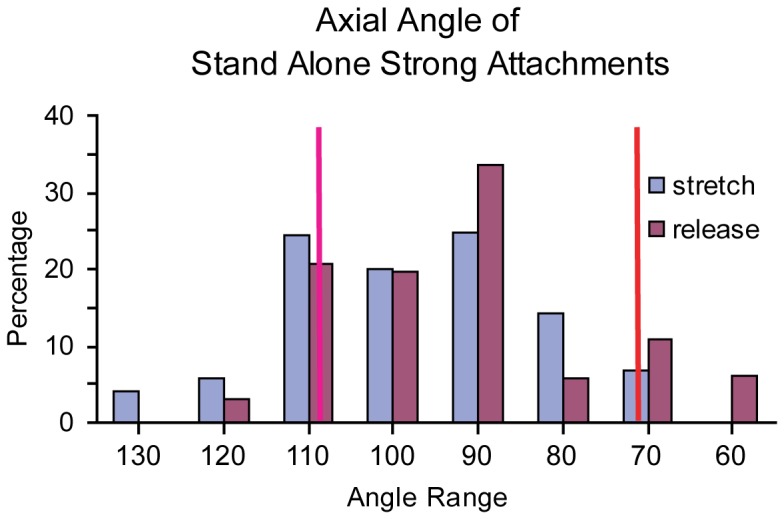
Axial angles of target zone stand alone strong attachments for str-HST and rls-HST. Bins are centered at 10° increments. Vertical red and magenta lines mark the lever arm angles of the two initial models (rigor and transition state). Mean values: 97° (str-HST) and 92° (rls-HST).

As a further effort to identify lever arm angle differences between stretch and release, we focused on the stand-alone one-headed strong attachments and excluded two-headed attachments or mask motifs, in which the lever arm angles are necessarily coupled, potentially obscuring the effects of stretch and release. Thus, the stand-alone attachments should be the best candidates to show the response to the length step direction with least ambiguity. However, between str- and rls-HST there is only a 5° difference in the mean and a 20° difference in the mode, indicating at most a 20° difference between the two groups (Fig. 10). That the observed lever arm angle differences are smaller than indicated by X-ray diffraction of frog muscle is likely due to the freezing impact times occurring later than ideal (1–2 ms after length step) and the ∼7-fold faster kinetics of the Phase 2 to Phase 3 transition expected in IFM at 23°C as compared to the ∼4°C of the classic frog muscle experiments.

### Azimuthal Distribution of Lever Arm Angles

Cylindrically averaged X-ray diffraction patterns cannot readily give information about the azimuthal changes in cross-bridge structure during contraction, and the only way to get azimuthal information is by imaging real cross-bridges in action as we have done. Azimuthally, the strong-binding cross-bridges sweep out a very broad range (∼140°) and are strongly biased anticlockwise (smaller azimuthal angles) with respect to the starting crystal structures regardless of stretch or release (Fig. 9B, D, & F, *purple*). Although the two crystal structures differ from one another by only 4° azimuthally [33], [36], very few of the observed strong-binding attachments have lever arms positioned azimuthally like the crystal structures, indicating that the crystal structures do not reflect the normal *in situ* constraints experienced by working IFM cross-bridges. Although the azimuthal ranges in all three states are similar, stretch and release has a large effect on the distribution of azimuthal angles of strong-binding attachments. In iso-HST, the mode is 60°, compared to ∼120° in the starting crystal structures. A step stretch brings the azimuthal mode to 90° and closer to the crystal structures. In contrast, a step release reduces the azimuthal mode to 30° indicating greater azimuthal alteration. The frequency of very small azimuthal angles seen in rls-HST is partly explained by the altered distribution of strong-binding attachments within the target zone. In rls-HST more attachments are on the Z-ward subunits J/K, which have less favorable azimuths for myosin attachment than do subunits H/I, relative to the cross-bridge origins on the adjacent thick filaments.

The azimuthal lever arm changes for weak-binding heads were much more modest and more symmetrical about the starting crystal structure. For the combined weak-binding heads from all three states (Fig. 9H, *red*), 64% fall within ±10° and 86% fall within ±20° of the starting myosin structure. The azimuthal mode for weak-binding bridges in iso- and rls-HST is 120° and represents attachments that did not need their lever arms azimuthally rotated away from the starting crystal structure in order to fit within the EM density. Even str-HST had a large fraction of weak attachments that could be fit without modifying the crystal structure.

## Discussion

When compared with iso-HST, a length perturbation resulted in changes to two important features of the structure: (1) the myosin head occupancy of actin subunits (or the associated Tn molecules) outside of the target zone and (2) the relative proportions of strong and weak-binding cross-bridges within the target zone. The highly restricted target zone of strong-binding heads in iso-HST is also found following a length perturbation but with a small expansion by one actin subunit following a stretch that added comparatively few strong-binding heads. Active myosin heads that attached to the target zone in iso-HST originated from a narrow zone on the thick filament which resulted in a wide and highly skewed distribution of azimuthal lever arm angles when compared to myosin head structures found in crystals. This feature is preserved following the length perturbation. The axial distribution of lever arm angles changed following the length perturbation by only a small amount but this change was consistent with expectations based on the direction of length perturbation and the time lapse before freezing.

### Changes in Actin Occupancy

Filament sliding caused by the length perturbation results in a small shift of the centroid of target zone labeling by strong-binding cross-bridges, while the overall position and span of the target zone within the 38.7 nm repeat remains largely unchanged and confined to just four actin subunits. The actin occupancy by strong attachments within the target zone shifted rather obviously toward the M-line for str-HST, and more subtly toward the Z-line for rls-HST (Fig. 4).

The distribution of weak-binding bridges is distinctly asymmetric with respect to the target zone. In all three HST states, on the M-ward side of the target zone, we frequently find weak binding bridges, which contact TM rather than actin. Z-ward of the target zone, we observe a near vanishing number of weak attachments on actin subunits L and M. With the exception of iso-HST, we observe few attachments on actins N and O, which are even further away. Thus, weak-binding bridges are favored in the direction toward which target zones move during sarcomere shortening and distinctly unfavored in the opposite direction. There may be a simple geometrical explanation to this observation based on the azimuthal origins of myosin heads and the actin azimuth, or it may be something else.

### Changes in Strong-Binding Cross-bridges

The character and distribution of the strong-binding cross-bridges changed significantly in response to the length perturbation. Two-headed cross-bridges were most common in iso-HST, but if the binding strength of the individual heads is taken into account two-headed, strong-binding cross-bridges were most prevalent in str-HST and least prevalent in rls-HST. If it is assumed that mixed two-headed attachments are a signature of isometric contraction, the fact that in str- and rls-HST, both heads of two-headed cross-bridges were always strong binding is consistent with tension at the instant of freezing having not yet developed to the new isometric level. The higher number of two-headed bridges in str-HST compared to rls-HST suggests that a step-stretch promotes two-headed bridge formation, while a step-release results in the loss of two-headed bridges. An increase in two-headed, strong-binding cross-bridges has previously been proposed to explain changes in the X-ray diffraction pattern of active, stretched vertebrate striated muscle fibers [5]. The limited size of the IFM target zone (two actin subunits) means that only a limited number of two-headed cross-bridges can form after a stretch and that these second heads must be partners of heads already attached to Z-ward target-zone actins and thus must attach to M-ward target zone actins in agreement with previous interpretations [5]. There is at the moment no earlier observation relating to the loss of two-headed bridges following a release.

Lever arm angles in two-headed cross-bridges are coupled. When two-headed bridges in the three states are compared, iso- and str- have similar average angles while for rls-HST, the average angles were significantly more toward rigor or the end of the power-stroke (Fig. 9), consistent with the direction of the length perturbation. The lever arm angles of two-headed str-HST bridges were not significantly different from iso-HST probably because they may be displaying an upper limit for bending specific to two-headed binding.

In str-HST, in addition to an increase in two-headed, strong-binding cross-bridges, a small fraction of strong-binding heads was found just M-wards of the target zone on actin subunit G (Fig. 4). Thus, the muscle’s response to lengthening during isometric contraction has a second component: enlargement of the target zone. Both responses would make sense as muscles are typically designed to generate sufficient tension to shorten when activated. If instead the muscle is forcibly lengthened while activated, it would need to increase the force it was producing by adding more strong-binding myosin heads either within the existing target zone, or by increasing the size of the target zone. The small number of weak-binding cross-bridges on the Z-ward side of the target zone suggest that few heads are poised to quickly bind in the target zone following a stretch making the best available alternative the addition of second heads or M-ward enlargement of the target zone. The observed effects of two-headed cross-bridges and target zone expansion might appear greater with a shorter time delay between the length step and the freezing impact.

### Changes to Pre-stroke Bridges

Dramatic differences in the number and distribution of target zone, weak-binding cross-bridges were observed after the length perturbation when compared with iso-HST. We previously described two types of weak-binding attachment within or near the target zone in iso-HST [25] based on whether the MD contacted actin or TM. Pre-stroke attachments contact actin and their confinement to the target zone supports their assignment as the weak-binding precursor to a strong-binding attachment. By contrast TM-bridges would probably have to bind a different actin subunit Z-ward of their current location to form strong attachments. After a length perturbation, either a stretch or a release, pre-stroke weak-binding attachments virtually disappear. This disappearance is not coincident with a large decrease in weak-binding, nonspecific attachments outside of the 4 target zone actins where the number of attachments remains roughly constant. Strong binding bridges change little.

This behavior can be *qualitatively* interpreted within the context of a comprehensive model of vertebrate muscle sarcomere dynamics that accounts for most of the known features of the contraction mechanism [37], [38]. To the extent that the mechanical kinetics and strain dependence of IFM are similar to vertebrate skeletal muscle, the occupancy of the weak and strong cross-bridge states and their changes upon quick stretches and releases can be considered. In this discussion, we use the kinetic scheme and strain dependent rate constants as illustrated in Figure 1 of [38]. In this comprehensive model the equivalent of pre-stroke, weak-binding bridges equilibrate with detached cross-bridges at rate constants of ∼50 s^−1^–200 s^−1^ (sum of k_34_ and k_43_) in the ±5 nm range of the target zone. These rates are comparable or somewhat faster than the time (8 ms) between the length steps and freezing in our experiment. Pre-stroke bridges in the model would have different outcomes for stretches and releases. For a 6–9 nm stretch, such as applied in our experiment, the detachment rate increases to 10^3^–10^4^ s^−1^ (k_43_), suggesting that pre-stroke weak attachments would all detach between stretch and freezing, as observed. Their displacement by >5 nm would place all of those initially bound to M-ward target zone actins (H and I) outside of the target zone on the M-ward side, from which position they would reform as TM-bridges not pre-stroke bridges. For a 6–9 nm release, the detachment rate also increases to 10^4^ s^−1^, but the conversion from weak to strong binding is even faster, >10^5^ s^−1^ (k_45_), indicating that most of the pre-stroke attachments would convert to strong binding ones. Those bound to Z-ward target zone actins that don’t convert to strong binding, would be displaced to a position opposite actin subunits L and M and not rebind. Thus pre-stroke attachments are expected to disappear for both directions of length changes.

For stretch, strong-binding bridges near the beginning of the working stroke can convert to weak attachments at 10^3^ s^−1^ and then detach (k_54_ followed by k_53_). If originally bound to M-ward target-zone actins, those that detach would not rebind as pre-stroke bridges but probably reform as TM-bridges outside the target zone. Strong-binding bridges toward the end of the working stroke are expected to tilt backwards at ∼10^3^ s^−1^ (k_75_). For release, strong-binding bridges complete their stroke and detach rapidly at 10^2^–10^3^ s^−1^ (k_81_). Not too many of them would reattach between the length step and freezing, requiring ATP hydrolysis at 50–100 s^−1^ (k_13_) and weak binding at 40 s^−1^–100 s^−1^ (k_34_). After the length step, many that detach would also find themselves outside the target zone and unable to rebind since weak actin attachments occur only rarely on actin subunits L and M located Z-ward of the target zone. The combination of these events and the conversion of weak to strong cross-bridges after release, seems to keep the number of strong-binding bridges roughly constant.

The Smith et al. model [37], [38] predicts very few weak binding cross-bridges outside of what we term as the target zone (±5 nm). The asymmetry of non-target zone weak attachments we observed indicates that no single equilibrium constant can adequately describe binding to individual actin subunits and that there is a significant geometrical or steric effect that influences weak binding, and thus the TM-bridges may re-equilibrate before the system is frozen.

TM-bridges are a novel type of cross-bridge. If they detach at a rate slower than 500 s^−1^ during the 2 ms release step, and instead diffuse along the thin filament, they would hold the cross-bridge in position for pre-stroke cross-bridge formation within the target zone (increasing k_34_). It is likely that these heads would also convert to strong binding before freezing. The possibility that TM-bridges may be comparatively unperturbed by the length step itself, changing instead to pre-stroke attachments which then convert rapidly to strong binding has not yet been considered in any kinetic scheme.

### Relationship to IFM Contraction

Many of the differences between str- and rls-HST may be understandable in terms of the typical shortening distance (TSD) of IFM. During oscillatory contractions, IFM fibers shorten much less than vertebrate striated muscle fibers. The amount of reported shortening varies between species but values ranging from 1–5% are typical [39], [40], [41], [42]. A shortening of 3% for *Lethocerus* IFM (half sarcomere length of 1.3 µm) equates to a filament sliding of 39 nm/half sarcomere, which matches the half repeat of the actin filament. Relaxed IFM fibers have very short I-bands compared to vertebrate striated muscle fibers consistent with short oscillatory contractions.

Wray’s match/mismatch hypothesis was offered as an explanation for stretch activation [43], but several aspects of cross-bridge structure and distribution found here in HST fibers can be explained within that context. Wray’s hypothesis states that as the filaments slide past each other the alignment of target zones relative to the thick filament origins of myosin heads changes from a region of best match with the shortest reach to a target zone to a region of worst match with the longest reach. The axial displacement between best match and worst match is approximately half of the actin crossover period, or 19.3 nm. Wray’s model did not define the portion of the thick filament surface from which myosin heads can attach to actin target zones. The crowns on the thick filament, which comprise the 4-fold symmetric origins of myosin heads every 14.5 nm axially, rotate in succession by 33.75° in a right-handed fashion [44]. The fact that mask motifs with strong-binding M-ward and Z-ward bridge pairs form on individual target zones means that the optimal myosin head origins must be contained within an arc of at least that angular width. As shown in iso-HST, this arc begins approximately at the interfilament axis.

A plausible length of the TSD in *Lethocerus* fibers can be derived from the helical spacings of the filaments and the length of the working stroke observed here. The sequence of events during shortening is shown in the animated Powerpoint S1. We assume that the pair of thick filaments (A and B) flanking a thin filament are axially aligned and with two crowns (1 and 2) positioned azimuthally so that heads from one myosin molecule in each crown can strongly bind their respective target zones (actin subunits H-K). We define the starting position of the TSD at z = 0 as the position where a strong-binding, target-zone attachment from crown 1 of thick filament B (crown 1B) can just be formed on M-ward target-zone actin subunit H. An axial translation of the thin filament by 2.75 nm enables a strong-binding attachment to be formed by a myosin head from crown 1A on target zone subunit I on the opposite side of the thin filament. If each head attached on the M-ward target zone actin subunits has a working stroke of 12 nm, actin subunit H can be transported 12+2.75 nm at which point myosin heads on crown 2 of thick filament B can attach actin H once the first heads are released. Even before this has occurred, second heads of myosins already attached by the first head to actins H and I can attach to Z-ward actin subunits J and K. Thus, a pair of axially aligned crowns interacting with the pair of M-ward target zone actin subunits move the thin filament more than 14.5 nm, which is enough to enable heads from crown 2 to attach actin. Myosin heads on crown 1 of both thick filaments attaching to Z-ward target zone actins do not contribute to the TSD. With a 12 nm working stroke, they merely sustain the shortening process. However, their contribution becomes more important if the working stroke is shorter than 12 nm. A myosin head from crown 2 of thick filament B can move target-zone actin subunit H a further 12 nm to position z = 14.5+12 nm. The second head from crown 2B attaching to Z-ward target zone actin J can move actin H a further 5.5 nm to z = 32 nm. Finally, the second head from crown 2A provides an additional 2.75 nm displacement through executing a working stroke on target zone actin K for a total displacement of 34.75 nm. Since myosin head origins and actin targets do not precisely align within the 116 nm axial period, some further extension of the shortening distance can be expected. Further shortening to 38.7 nm would allow these events to repeat on the next target zone, but this may not be consistent with shortening deactivation.

This TSD predicts that the target zone actin occupancy would change as the muscle shortens. At the beginning of the TSD, the first strong-binding attachments would occur on the M-ward actins, H and I, of the target zone. As the muscle shortens further and the match between origins and actin targets improves to its maximum, the peak of occupancy for strong-binding attachments would shift more toward the center of the target zone with equal numbers of M-ward and Z-ward target-zone attachments. This is probably the position that produces maximum isometric tension. Further shortening reduces the match between myosin head origins and targets, with the peak of occupancy shifting toward the Z-ward actins, J and K. Eventually the target zone would move beyond the region of match to a fully mismatched position.

Stretching the muscle is essentially working backwards through the sequence; hence the peak of occupancy for strong-binding attachments shifts toward the distribution found at the beginning of the contraction, i.e. toward the M-line side of the target zone as observed here in str-HST. The opposite, a quick release, allows the muscle to shorten, thereby shifting the peak of occupancy toward the end of the TSD and thus toward the Z-line. That the centroid never shifted completely from M-ward to Z-ward actin subunits or vice versa is explicable by the relatively short length steps, 6–9 nm/half sarcomere, used in the present experiments and the possibility that the isometric tension which formed the starting point for the length perturbation was trapped near the center of the best match zone.

This model incorporates a relay mechanism in which the target zone is “relayed” from one 14.5 nm crown of myosin heads to the next, with myosin heads on the next M-ward crown attaching before the ones in the preceding, Z-ward crown detach. Such a mechanism was proposed when mask motifs were first identified in IFM treated with AMP-PNP [45].

In the TSD model described above, the first myosin heads (crown 1) to bind the target zone would originate azimuthally ∼34° from the inter-filament axis, which is a position least optimal for strong binding to actin. However, the first actin subunit on the M-line side of the target zone has a more optimal azimuth for strong binding than the Z-ward actin subunit, which would help ameliorate this disadvantage. Myosin heads originating from the preceding crown (crown 0) would have no myosin head origins that fall within the allowed region. As filament sliding progresses and the target zone is brought within reach of crown 2, the accessible myosin from this crown would have a much more favorable azimuth for target zone binding. The next crown after this (crown 3) would have no myosin head origins within the observed 34° range. Not all target zones on a thin filament would be accessible by myosin heads originating from two successive crowns and this may explain why not all cross-bridges are part of mask motifs near the isometric tension level.

TM bridge attachments were altered significantly following the length perturbation consistent with the hypothesized relay sequence. We consider these weak-binding bridges to be candidates for strong-binding bridges once the target zone moves closer to them. Because of their location M-ward of the target zone and the right-handed twist of the thin filament, when they swing outward, they strike TM rather than actin and thus do not progress further toward strong binding. Their numbers are much higher in str-HST and are lowest in rls-HST. The difference between str- and rls-HST TM-bridges can be understood as an effect of the length perturbation either advancing (rls-HST) or reversing (str-HST) the shortening events. Early in the sequence, it would be advantageous to have myosin heads, meaning TM-bridges, lying in wait for the target zone to move toward them so that they could bind strongly to continue the shortening process. Conversely, toward the end of the shortening cycle, there is less need for such attachments as it is desirable for tension to drop, a phenomenon known as shortening deactivation.

Stretching the muscle moves the target zone toward a position earlier in the TSD facilitating formation of TM-bridges from those target-zone heads that detach as tension drops. Releasing the muscle moves the target zone toward the end of the shortening cycle thereby “consuming” the available TM-bridges by presenting them with a favorably placed target zone to which they can bind strongly. The necessity for TM-bridges waiting to bind target zone actins to sustain further shortening would be expected to diminish closer to the end of the sequence.

Perhaps even more surprising is the broad azimuthal distribution of strong-binding attachments. Compared to the two crystal structures used as initial atomic models, the lever arm azimuths of strong-binding attachments are strongly skewed in the anticlockwise direction (looking Z-wards) thus confirming the observation made previously for iso-HST [25]. Weak-binding attachments, on the other hand, are spread roughly symmetrically about the initial models (Fig. 9). The strong anticlockwise spread of the lever arms was previously observed in quasiatomic models of rigor IFM both when swollen in low ionic strength [46] and when subjected to a stretch [47], but its significance could not be evaluated because there was no control group of either unattached or weakly attached myosin heads and at the time there was no acto-S1 structure, which later showed that IFM myosin S1 is almost indistinguishable from other acto-S1 structures [48]. For the present results the weak-binding attachments serve as a control group. We do not believe that the wide azimuthal range is an error in either classification or model building although the range may have been expanded by 10°–20° due to section compression. Thus, we think the wide range represents real structure and have suggested that it may reflect the weak to strong transition [25], an azimuthal component to the working stroke [49] or both.

Could the azimuthal changes in the lever arm reflect storage of elastic energy? Were this so, str-HST would be predicted to show a distribution biased toward smaller angles (i.e. more anticlockwise and more distorted) since elastic energy has been added to the fibers by means of the stretch. What little bias there is for str- compared to iso-HST is toward higher angle (more clockwise), just the opposite of expectation. The orientations in rls-HST, in which the release has dissipated elastic energy, are also the opposite of expectation since their distribution is biased toward smaller angles.

It seems more likely that the azimuthal changes might act as a trigger for promoting the conversion to strong binding.

### Conclusion

Compared to low angle X-ray experiments, the other popular technique to study the muscle cross-bridge structures *in situ*, EM has the advantage of direct visualization, which makes it possible to identify structure variations within an ensemble, even for sparsely populated forms, instead of the average structure. Our analysis allows us to locate and enumerate particular cross-bridge forms. The mechanical traces indicate that after the length perturbation, both str- and rls-HST were well on their way toward establishing a new isometric tension level for the new sarcomere length, a conclusion born out by the structures observed. That weak binding, pre-power-stroke attachments disappeared after the length step is consistent with expectations based on the rates of conversion from weak to strong binding. Myosin head binding throughout the 38.7 nm repeat is indicative of a myosin head relay mechanism that occurs over small changes in sarcomere length.

## Materials and Methods

### Collection of Live Giant Waterbugs

No specific permits were required for the collection of live giant waterbugs of species *Lethocerus indicus*. Live giant waterbugs were purchased by an agent from local farmers in Thailand where they are collected as a food item in the local diet. *Lethocerus indicus* is not on the global endangered species list either globally or locally in Thailand (http://www.earthsendangered.com/list.asp). Live giant waterbugs were imported into the USA under a USDA permit PPQ26. Giant waterbugs are considered to offer no agricultural threat or interest in the USA.

### Rapid Freezing and Freeze Substitution

Single fibers, diameter 60–70 µm, length 12–16 mm, were dissected cold and glued to transducer pins with no stretching, keeping 5–6 mm clear between glued ends for slam-freezing. Rapid freezing with simultaneous monitoring of fiber tension up to the moment of freezing impact was performed on a Heuser Cryopress [50]. Specific modifications made to the freezing head for this work have been described in detail, as have specifics of the specimen manipulation prior to and subsequent to freezing [22], [51]. The fastest length perturbation that could be applied with this system was 2 ms duration due to the amount of damping that could be applied to the bimorph transducer.

### Electron Tomography

Details of all the data collection and subvolume analysis have been described [24]. Three pairs of tilt series were collected and three dual axis tomograms were reconstructed for both str-HST and rls-HST. A total of 1157 cross-bridge repeats were extracted for alignment and MDA from the three str-HST tomograms. A total of 782 cross-bridge repeats were extracted for alignment and MDA from the three rls-HST tomograms.

### Repeat Subvolume Processing

Subvolume processing for str- and rls-HST followed the same general procedures used for iso-HST [24], [25]. Repeat sub-volumes were centered on the actin target zones spaced 38.7 nm apart axially and contained a 60.7 nm axial length of the actin filaments, their bound cross-bridges and adjacent thick filament segments. All subvolumes were aligned onto the actin filament using multireference alignment and classification. The alignment and classification masks were the same except for the edge apodization applied to the alignment mask. Both str- and rls-HST repeats were aligned in the final step to the same thin filament reference to simplify quasiatomic model building.

We performed 12 independent applications of MDA and classification using 12 different masks to define specific regions. To identify the major cross-bridge forms in and around the target zone, two primary masks covered the space between thick and thin filaments over an axial distance corresponding to six actin subunits on the left or right side respectively. All quasiatomic cross-bridge models were built from these two classifications, which we refer to as “primary classes.” We generated 20 primary class averages from each side of rls-HST, and 30 primary class averages from each side of str-HST as permitted by the greater number of str-HST repeats available. Four additional masks were used for the troponin region, four masks were specific for actin subunits outside of the target zone and two masks were used for the surface of the thick filament. Class averages from the primary mask and Tn region classifications were subsequently reassembled to make composite class averages [24]. All the structures described in this report come from these reassembled repeats.

### Quasiatomic Models

Quasiatomic models were built in a hierarchical fashion [24]. The thin filament atomic model was fit to the global average of all repeats. The F-actin atomic model based on PDB - 1M8Q was constructed of 16 G-actin monomers built with the 28/13 helical structure appropriate to IFM thin filaments [52]. This enabled placement of two pairs of Tn models [53] one pair on each end of the filament. Although not resolved in the reconstruction, the TM location is important for structure interpretation. Thus, TM in the high [Ca^2+^] position [54] was built into the thin filament along with a fragment sufficient to cover the extra pair of Tn and actin subunits.

For cross-bridges whose lever arm orientations were angled toward the rigor configuration, we used an atomic model adapted from the Holmes et al. rigor acto-S1 complex [33] (available at ftp://149.217.48.3/pub/holmes). For cross-bridges whose lever arms appeared to be perpendicular to the thin filament or were angled opposite to rigor, we used the transition state of scallop myosin S1, PDB - 1DFL [36], after aligning its MD to the Holmes rigor MD position.

To distinguish weak from strong binding we evaluated the fitting of the MD first. If the starting model’s MD fit the density without modification, it was kept in this effectively strong-binding configuration, and only the lever arm adjusted using as pivot points residues 710, 780 and 806. If the MD required movement, the attachment was considered weak and all subsequent manipulations were done using the scallop S1 structure. For these weak-binding bridges, the entire S1 structure was first moved as a single rigid body; its lever arm position was then adjusted if necessary using residues 706, 775 and 806 as pivot points. Manual fitting was done using the X-ray crystallography model fitting program O [55].

Models were built separately into those classes obtained by the left-side and right side primary mask class averages. These models were then combined as necessary to produce all of the complete quasiatomic models. Verification of the location of the C-terminus of the myosin heads was done by computing separately class averages using the mask specific for the surface of the thick filament backbone, and readjusting the lever arm if indicated [24]. We checked the origins of cross-bridges against the original raw repeats if there was any remaining doubt.

All figures and movies of quasiatomic models were made in chimera [56].

## Supporting Information

Movies S1 This movie corresponds to repeat rls-22 shown in Figure 7. It shows on the left a mask motif structure composed of a pair of strong binding cross-bridges. A single headed cross-bridge is shown on the right. All the cross-bridges are strong binding. A Tn bridge is present on the lower left side of the thin filament, but without an associated atomic model. The coloring scheme is the same as for Figure 7. Movie made in chimera [56].
(MOV)

Movies S2 This movie corresponds to repeat rls-47 shown in Figure 7. It shows a pair of single headed, strong binding cross-bridges in the target zone and a Tn bridge on the left side, but without an associated atomic model. The coloring scheme is the same as for Figure 7. Movie made in chimera [56].
(MOV)

Movies S3 This movie corresponds to repeat rls-65 shown in Figure 7. It shows a double-headed cross-bridge on the left side and a single-headed cross-bridge on the right side, all with strong binding myosin heads. A Tn bridge is shown in the lower right hand side but without an associated atomic model. The coloring scheme is the same as for Figure 7. Movie made in chimera [56].
(MOV)

Movies S4 This movie corresponds to repeat str-25 shown in Figure 7. It shows a pair of single-headed cross-bridges in the target zone and a TM bridge above the target zone on the left hand side. The coloring scheme is the same as for Figure 7. Movie made in chimera [56].
(MOV)

Movies S5 This movie corresponds to repeat str-127 shown in Figure 7. It shows a pair of single headed cross-bridges but the one on the left is bound to out-of-target zone actin subunit G. This is the only example of an out-of-target-zone, strong-binding cross-bridge found among any of the str- or rls-HST class averages. The coloring scheme is the same as for Figure 7. Movie made in chimera [56].
(MOV)

Movies S6 This movie corresponds to repeat str-128 shown in Figure 7. It shows a single-headed, strong-binding cross-bridge on the left and a double-headed, strong-binding cross-bridge on the right. The coloring scheme is the same as for Figure 7. Movie made in chimera [56].
(MOV)

Powerpoint S1 This file illustrated the typical shortening distance as described in section on the Relationship to IFM contraction. The individual slides of the Powerpoint file are described within the file itself.
(PPT)
