# Supplementary material for: Structural Changes in Isometrically Contracting Insect Flight Muscle Trapped following a Mechanical Perturbation
Source: PLoS One. 2012 Jun 25;7(6):e39422. doi: 10.1371/journal.pone.0039422 (PMC3382574; doi:10.1371/journal.pone.0039422)
Supplement: Powerpoint S1 — This file illustrated the typical shortening distance as described in section on the Relationship to IFM contraction. The individual slides of the Powerpoint file are described within the file itself. (PPT) [file pone.0039422.s007.ppt]

## Slide 1
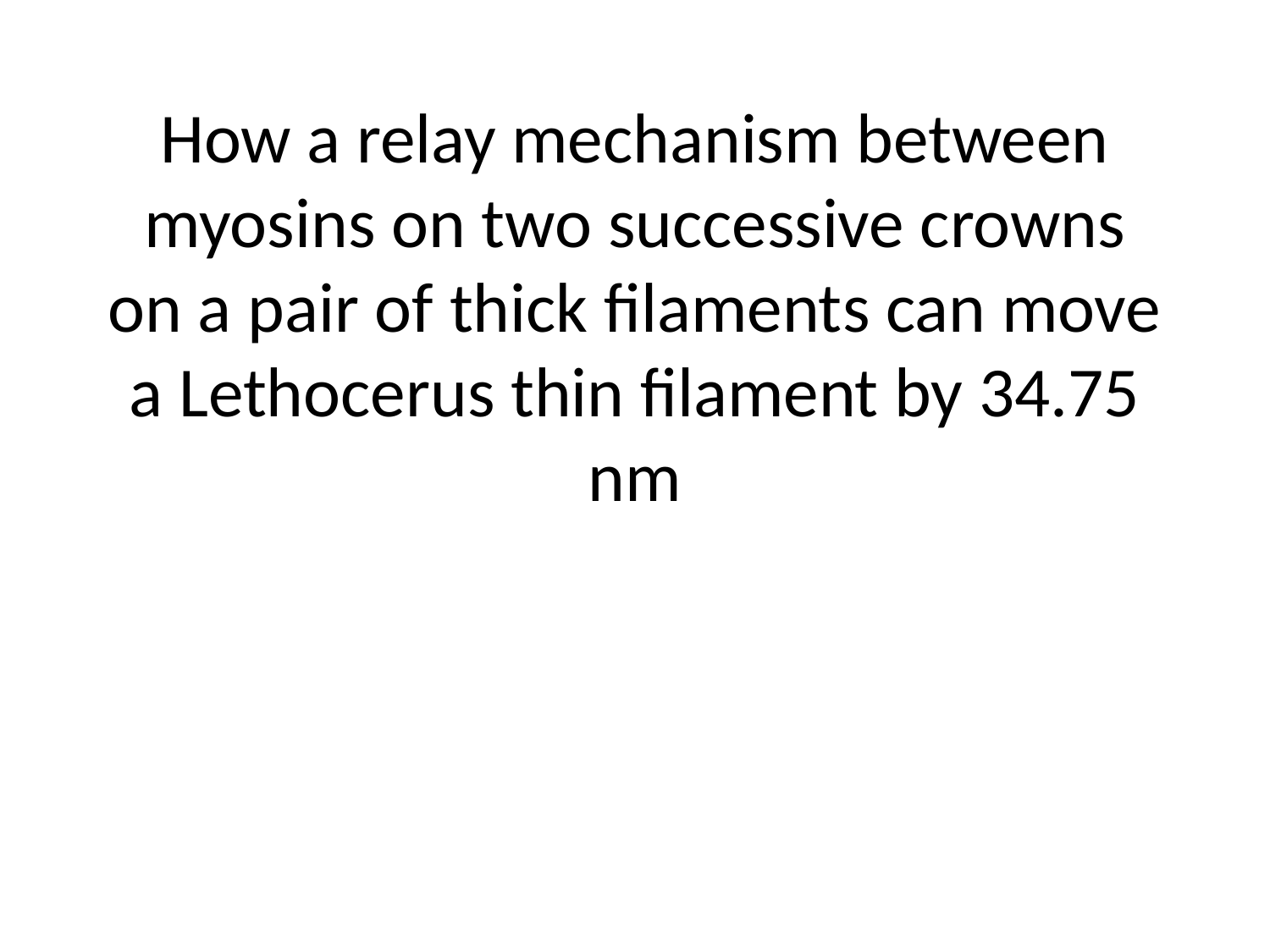

# How a relay mechanism between myosins on two successive crowns on a pair of thick filaments can move a Lethocerus thin filament by 34.75 nm

## Slide 2
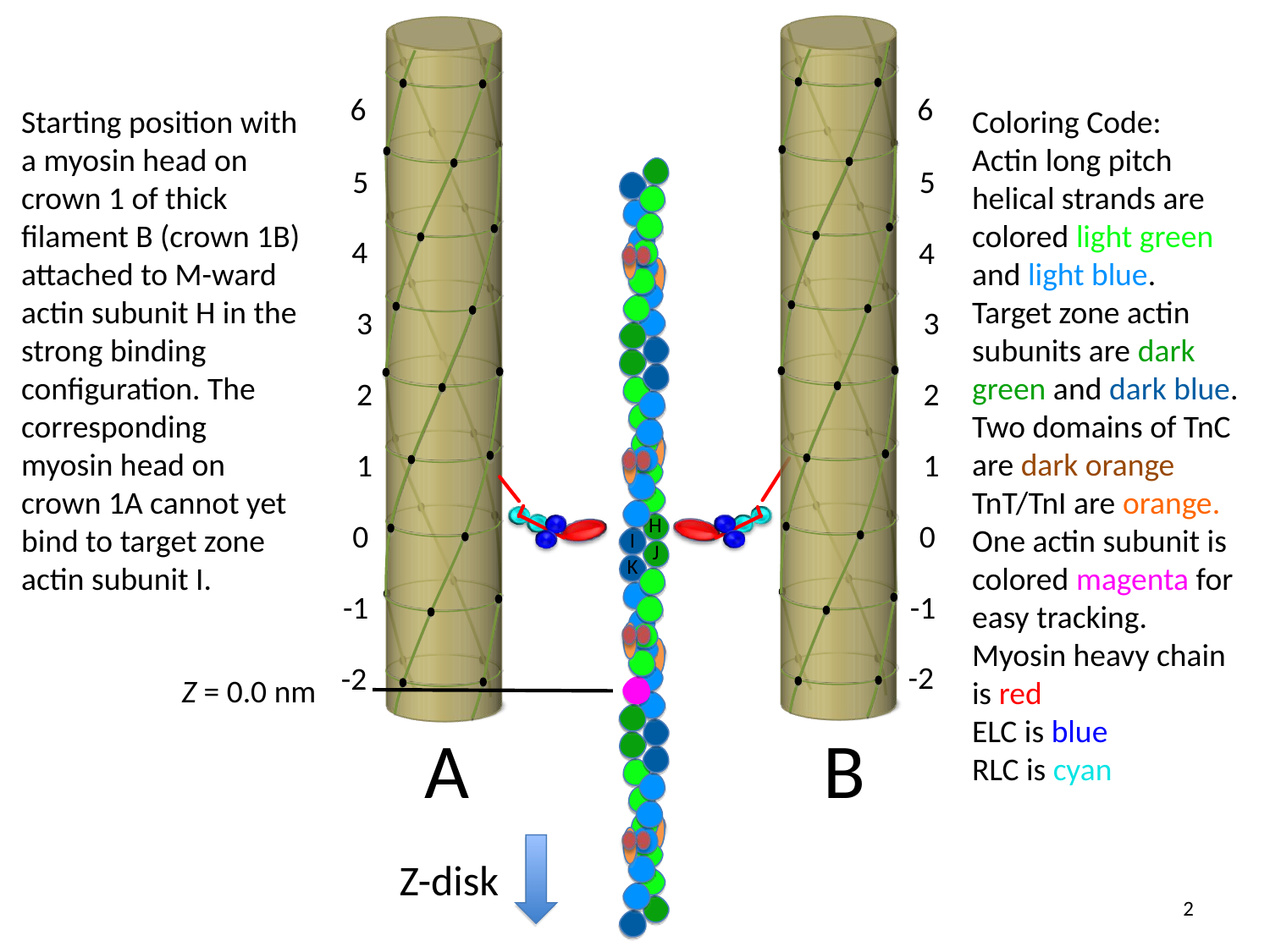

6
5
4
3
2
1
0
-1
-2
6
5
4
3
2
1
0
-1
-2
Starting position with a myosin head on crown 1 of thick filament B (crown 1B) attached to M-ward actin subunit H in the strong binding configuration. The corresponding myosin head on crown 1A cannot yet bind to target zone actin subunit I.
Coloring Code:
Actin long pitch helical strands are colored light green and light blue.
Target zone actin subunits are dark green and dark blue.
Two domains of TnC are dark orange
TnT/TnI are orange.
One actin subunit is colored magenta for easy tracking.
Myosin heavy chain is red
ELC is blue
RLC is cyan
H
I
J
K
Z = 0.0 nm
A
B
Z-disk
<number>

## Slide 3
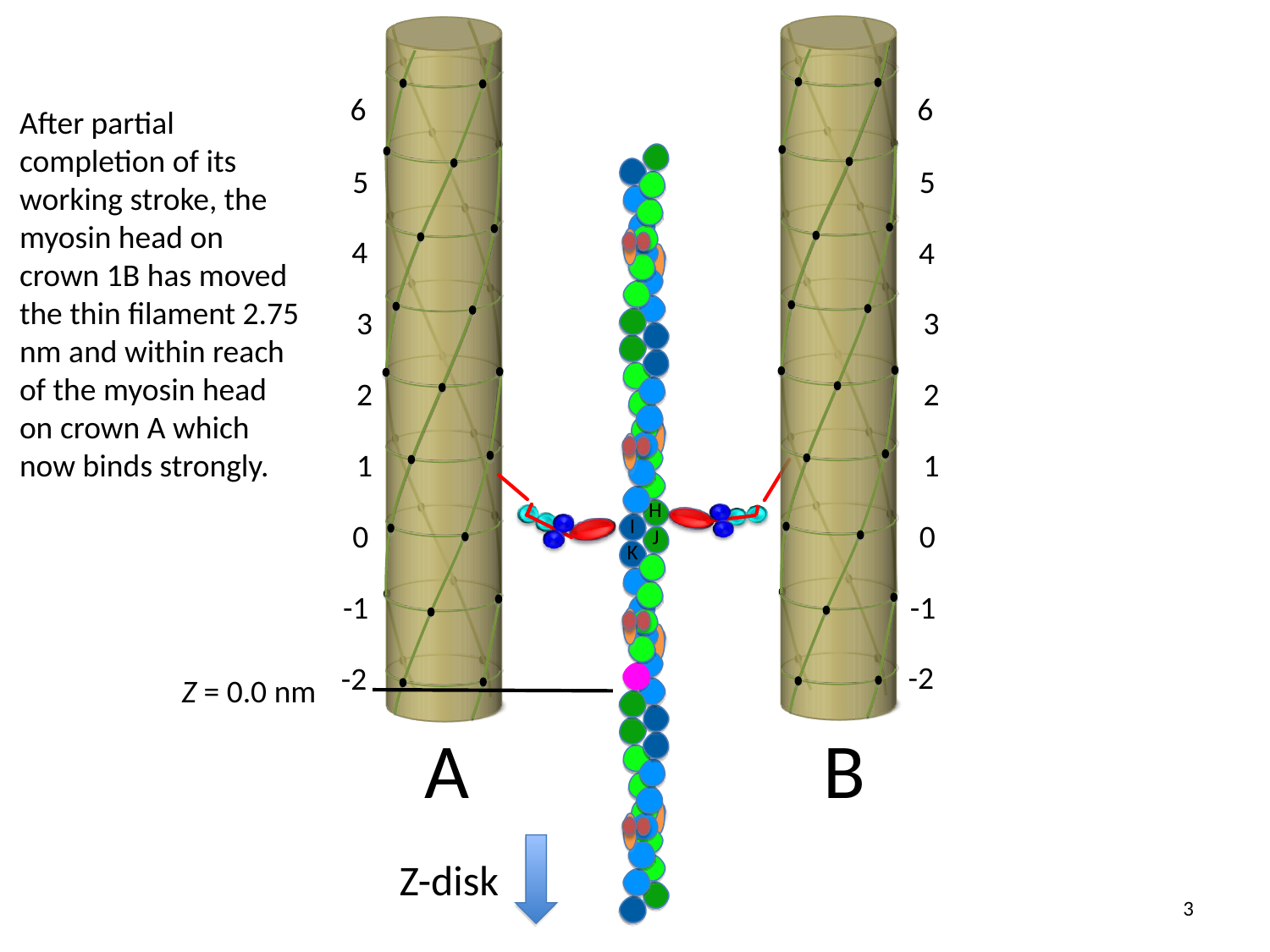

6
5
4
3
2
1
0
-1
-2
6
5
4
3
2
1
0
-1
-2
After partial completion of its working stroke, the myosin head on crown 1B has moved the thin filament 2.75 nm and within reach of the myosin head on crown A which now binds strongly.
H
I
J
K
Z = 0.0 nm
A
B
Z-disk
<number>

## Slide 4
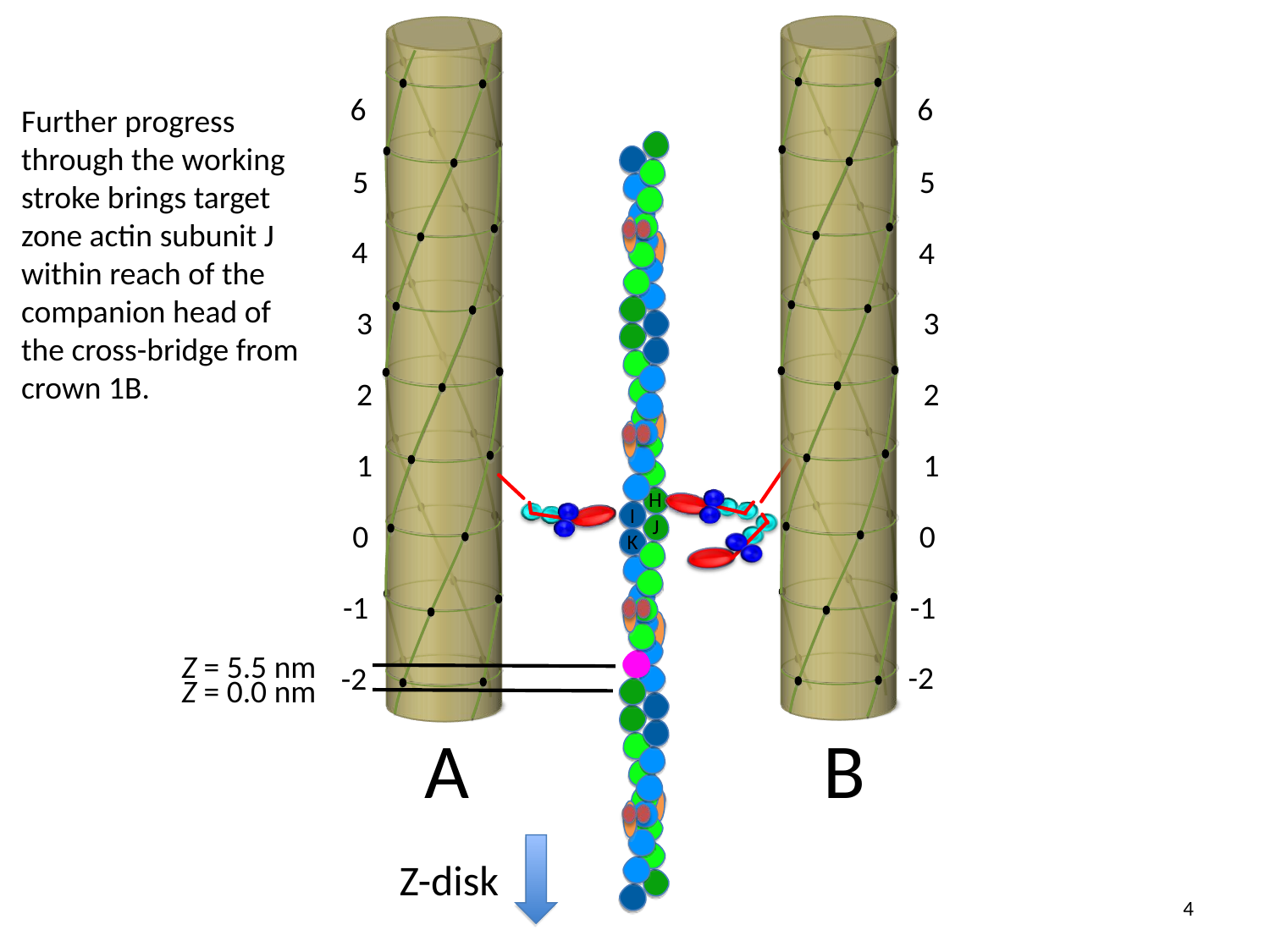

6
5
4
3
2
1
0
-1
-2
6
5
4
3
2
1
0
-1
-2
Further progress through the working stroke brings target zone actin subunit J within reach of the companion head of the cross-bridge from crown 1B.
H
I
J
K
Z = 5.5 nm
Z = 0.0 nm
A
B
Z-disk
<number>

## Slide 5
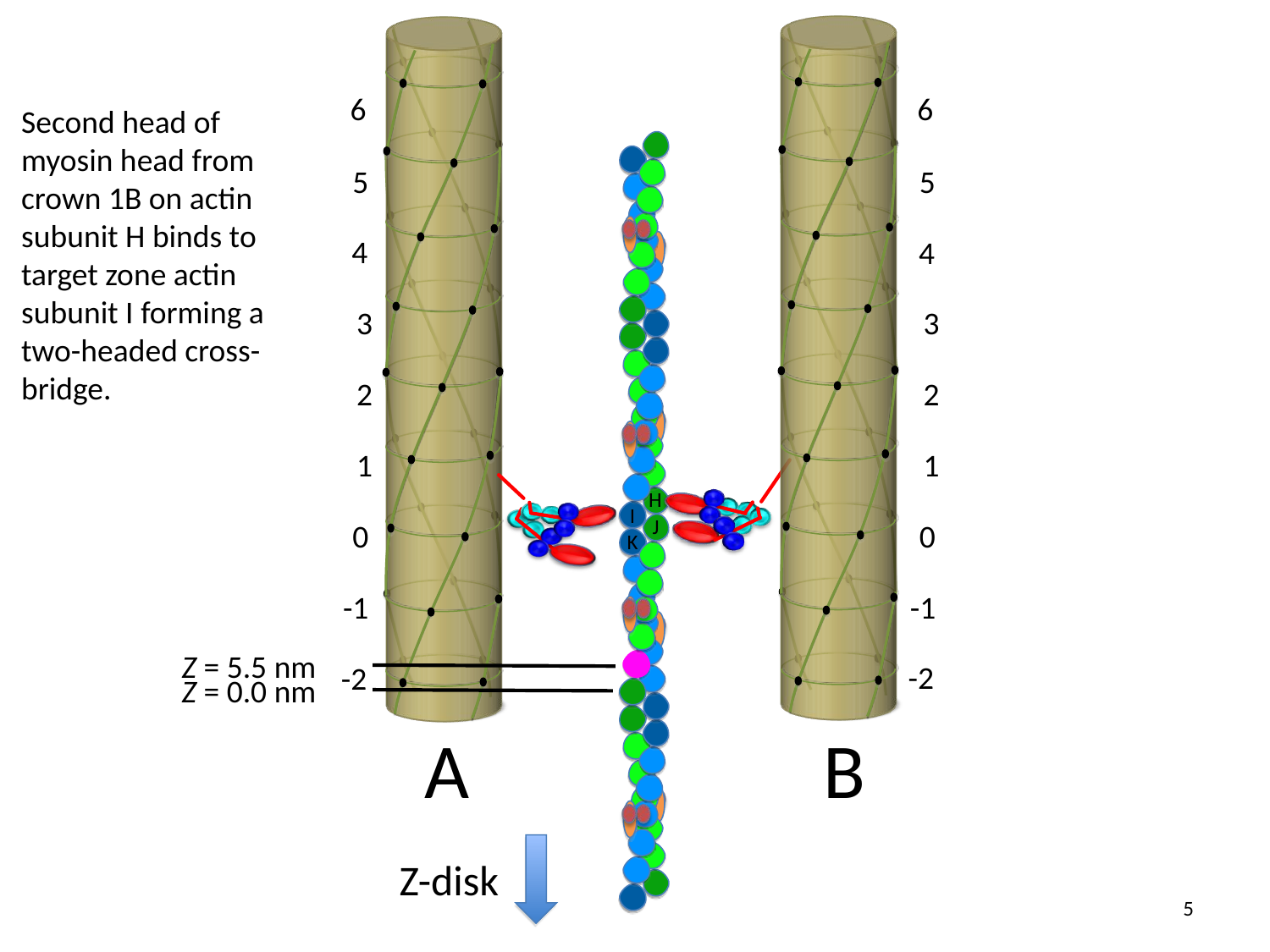

6
5
4
3
2
1
0
-1
-2
6
5
4
3
2
1
0
-1
-2
Second head of myosin head from crown 1B on actin subunit H binds to target zone actin subunit I forming a two-headed cross-bridge.
H
I
J
K
Z = 5.5 nm
Z = 0.0 nm
A
B
Z-disk
<number>

## Slide 6
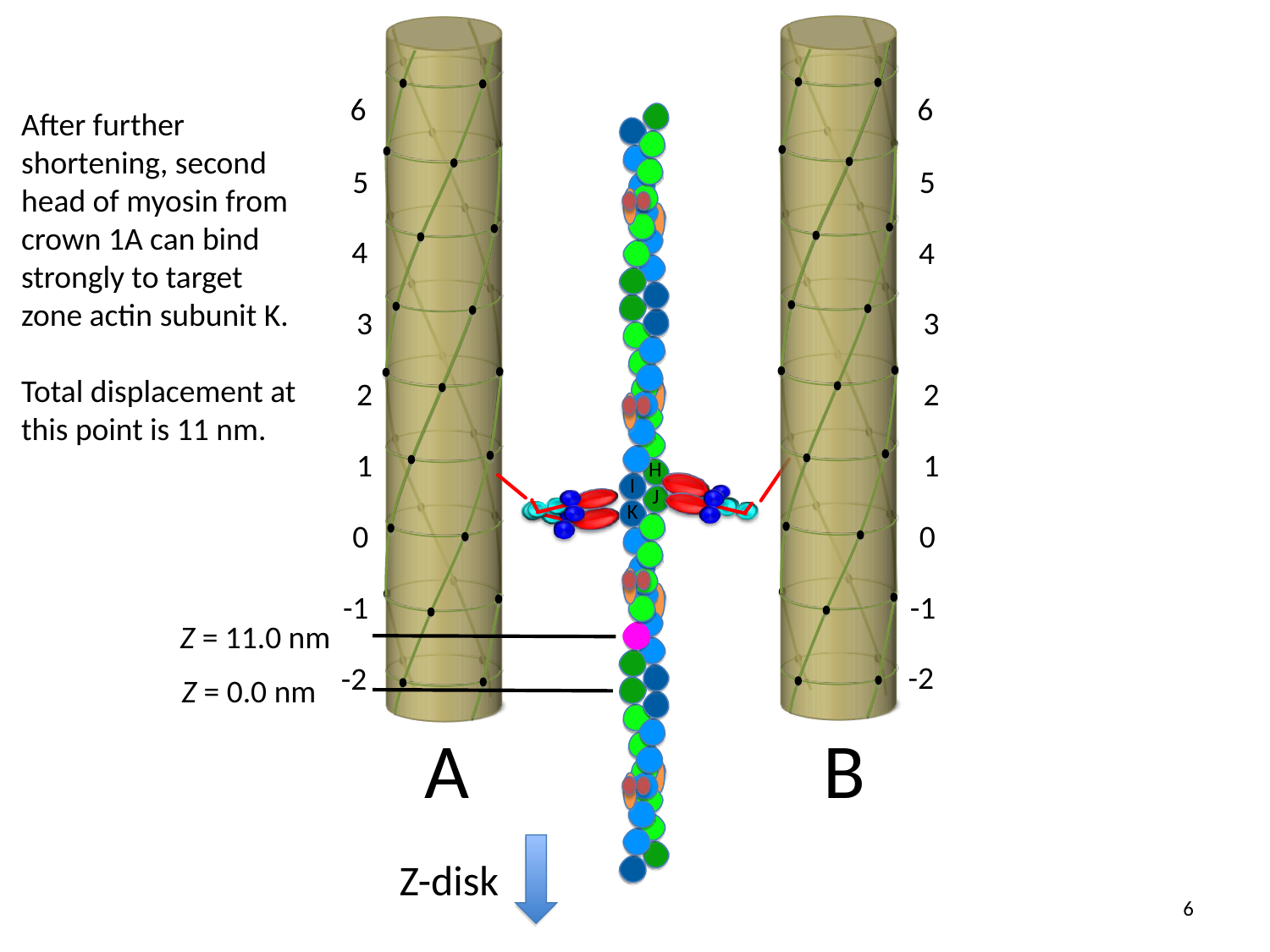

6
5
4
3
2
1
0
-1
-2
6
5
4
3
2
1
0
-1
-2
After further shortening, second head of myosin from crown 1A can bind strongly to target zone actin subunit K.
Total displacement at this point is 11 nm.
H
I
J
K
Z = 11.0 nm
Z = 0.0 nm
A
B
Z-disk
<number>

## Slide 7
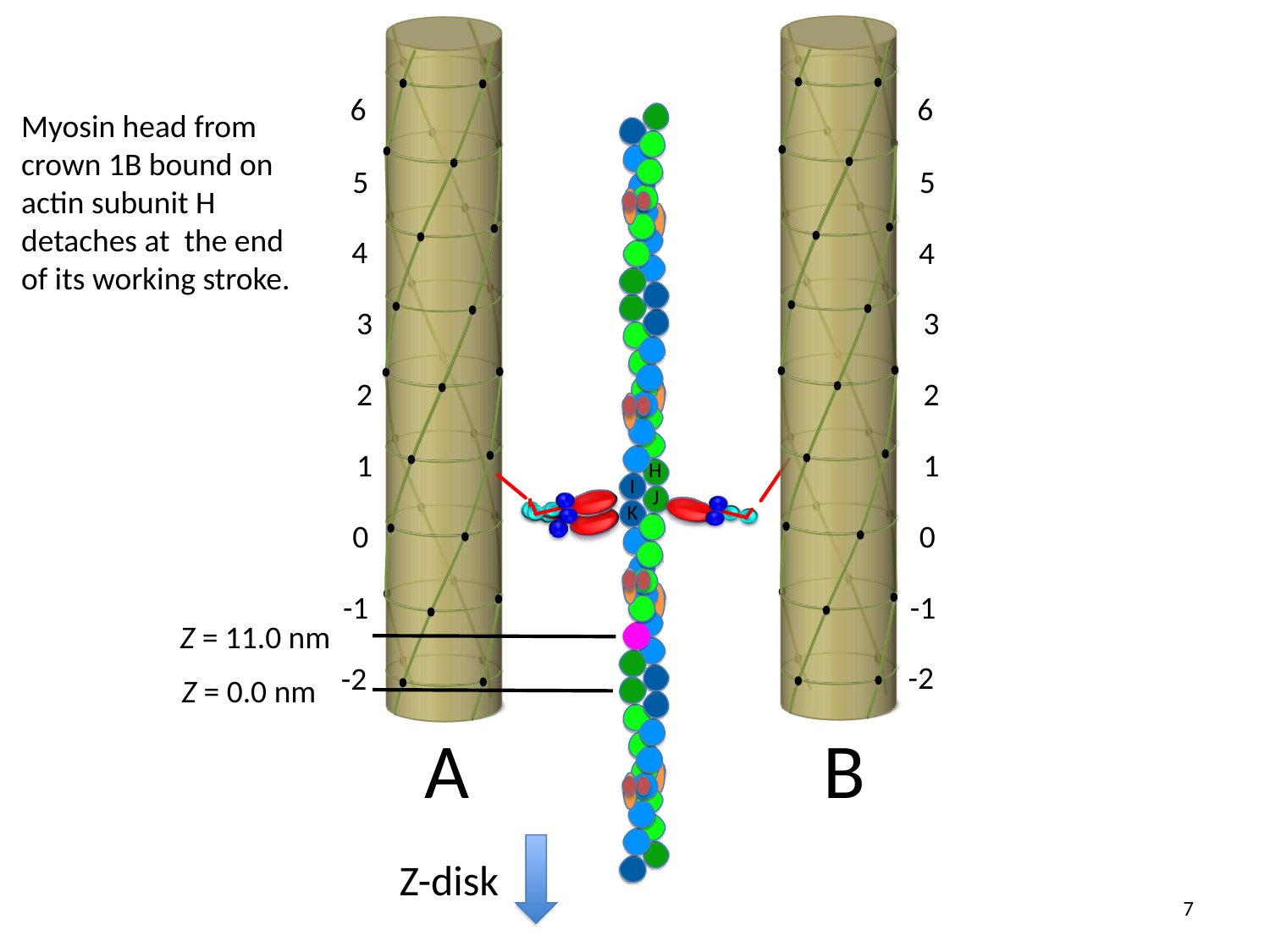

6
5
4
3
2
1
0
-1
-2
6
5
4
3
2
1
0
-1
-2
Myosin head from crown 1B bound on actin subunit H detaches at the end of its working stroke.
H
I
J
K
Z = 11.0 nm
Z = 0.0 nm
A
B
Z-disk
<number>

## Slide 8
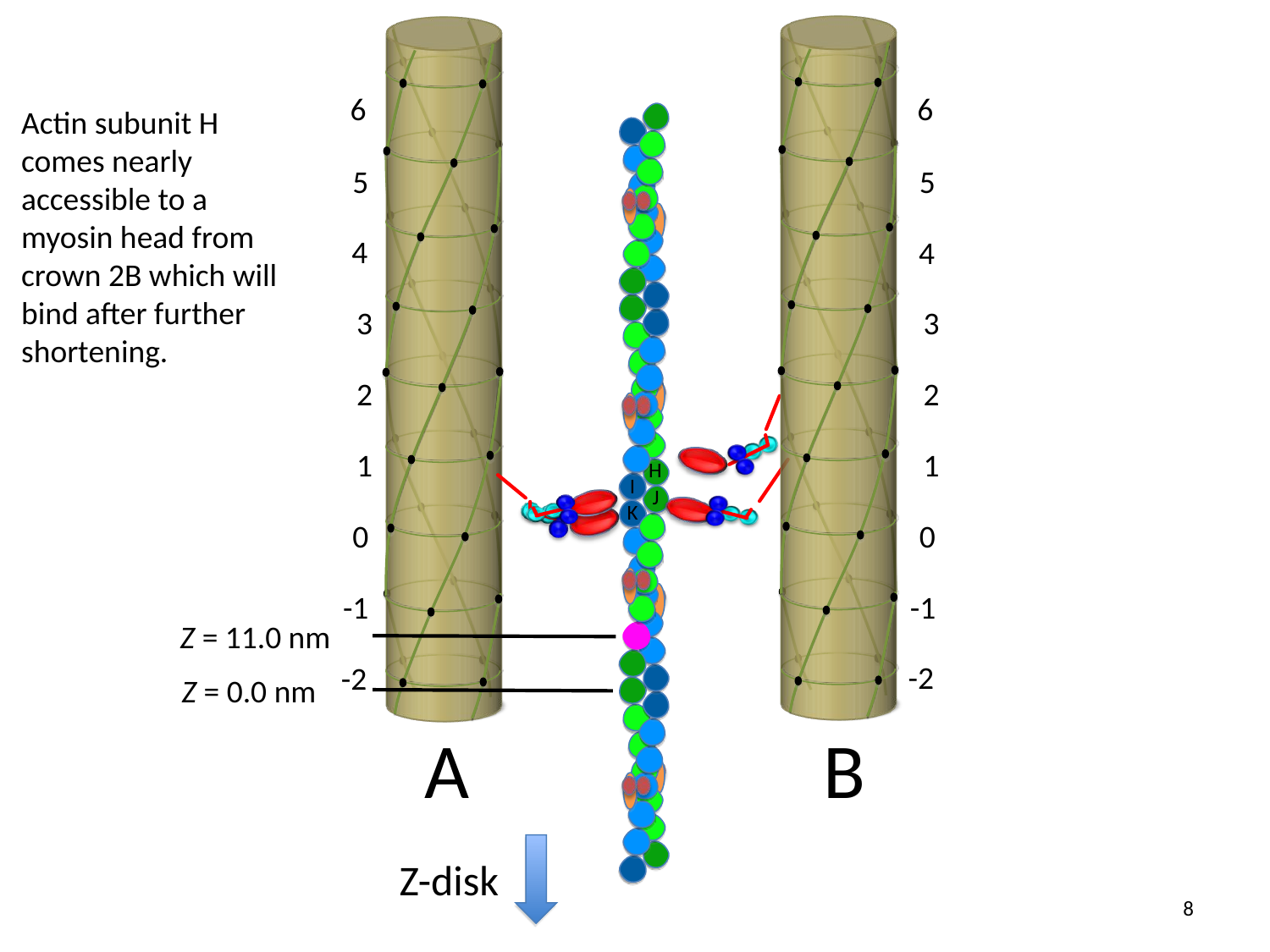

6
5
4
3
2
1
0
-1
-2
6
5
4
3
2
1
0
-1
-2
Actin subunit H comes nearly accessible to a myosin head from crown 2B which will bind after further shortening.
H
I
J
K
Z = 11.0 nm
Z = 0.0 nm
A
B
Z-disk
<number>

## Slide 9
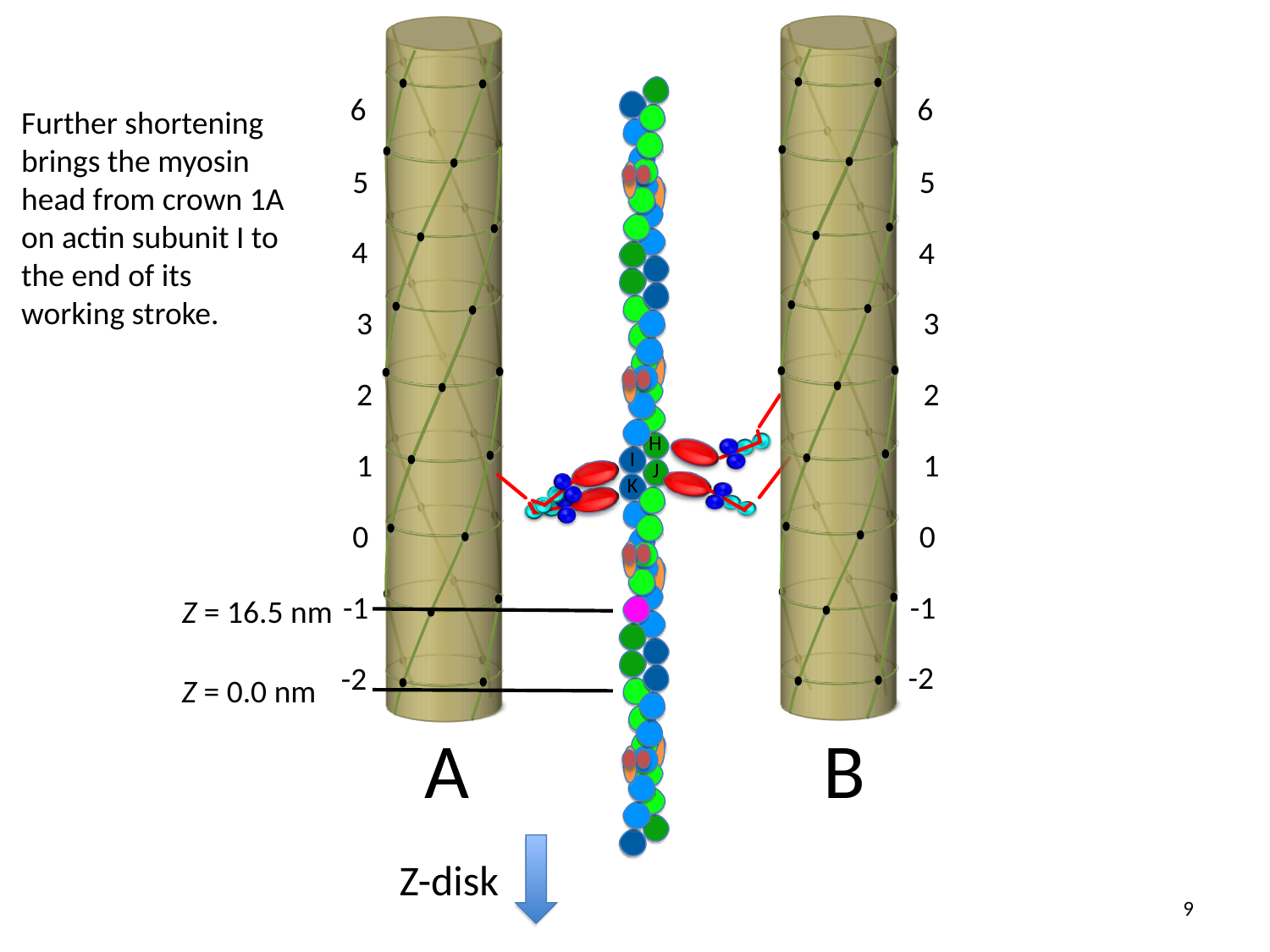

6
5
4
3
2
1
0
-1
-2
6
5
4
3
2
1
0
-1
-2
H
I
J
K
Further shortening brings the myosin head from crown 1A on actin subunit I to the end of its working stroke.
Z = 16.5 nm
Z = 0.0 nm
A
B
Z-disk
<number>

## Slide 10
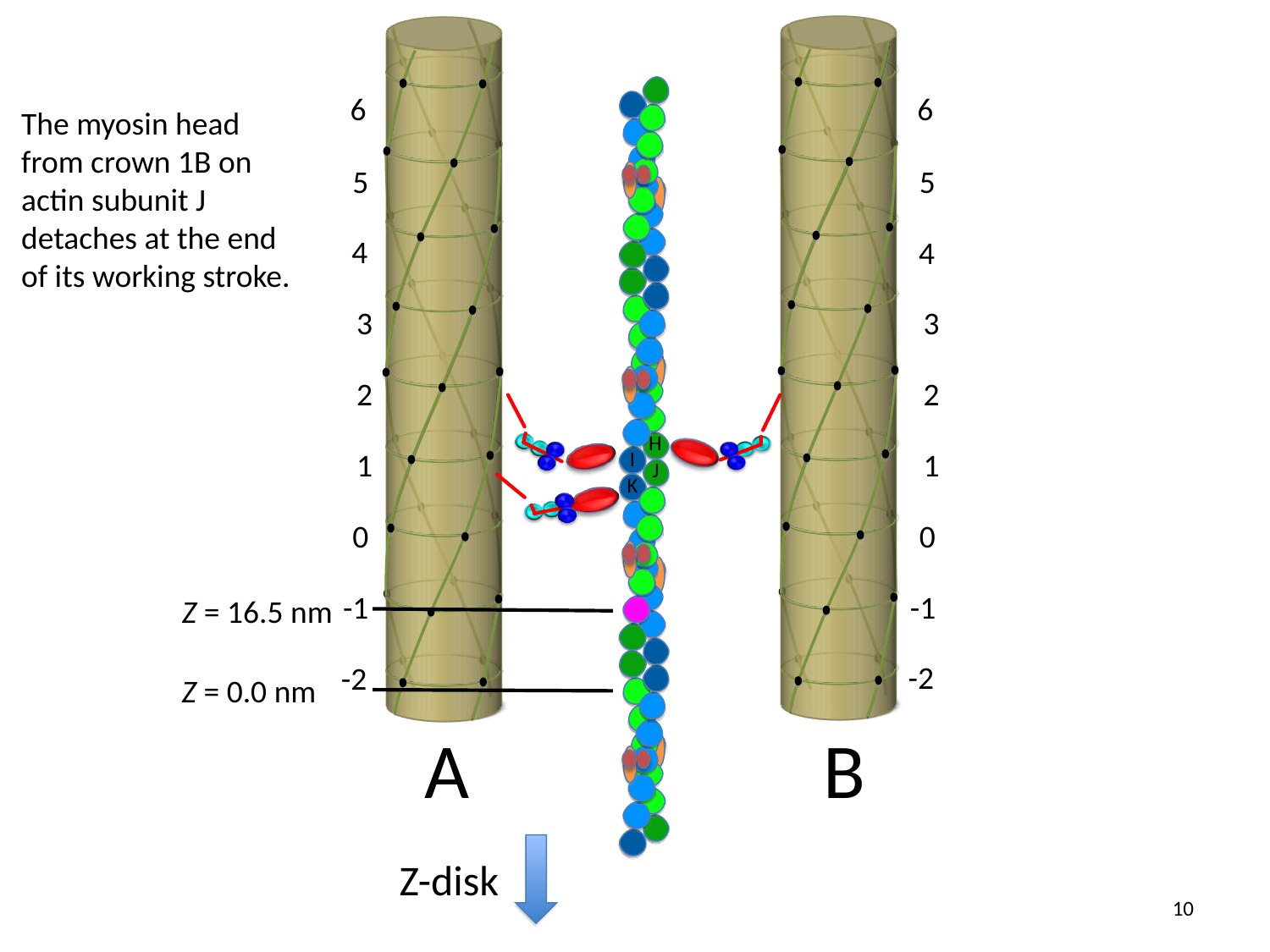

6
5
4
3
2
1
0
-1
-2
6
5
4
3
2
1
0
-1
-2
H
I
J
K
The myosin head from crown 1B on actin subunit J detaches at the end of its working stroke.
Z = 16.5 nm
Z = 0.0 nm
A
B
Z-disk
<number>

## Slide 11
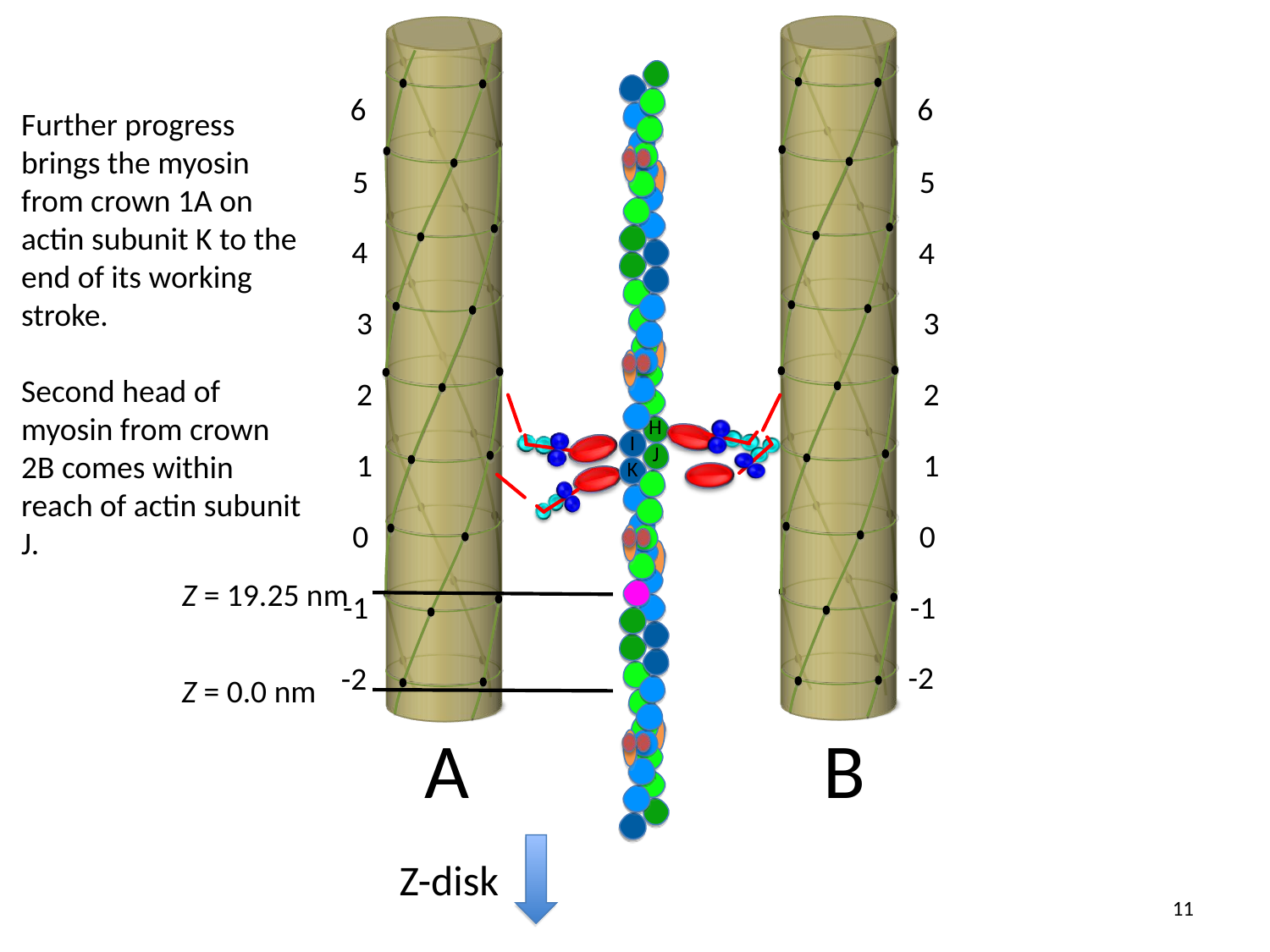

6
5
4
3
2
1
0
-1
-2
6
5
4
3
2
1
0
-1
-2
H
I
J
K
Further progress brings the myosin from crown 1A on actin subunit K to the end of its working stroke.
Second head of myosin from crown 2B comes within reach of actin subunit J.
Z = 19.25 nm
Z = 0.0 nm
A
B
Z-disk
<number>

## Slide 12
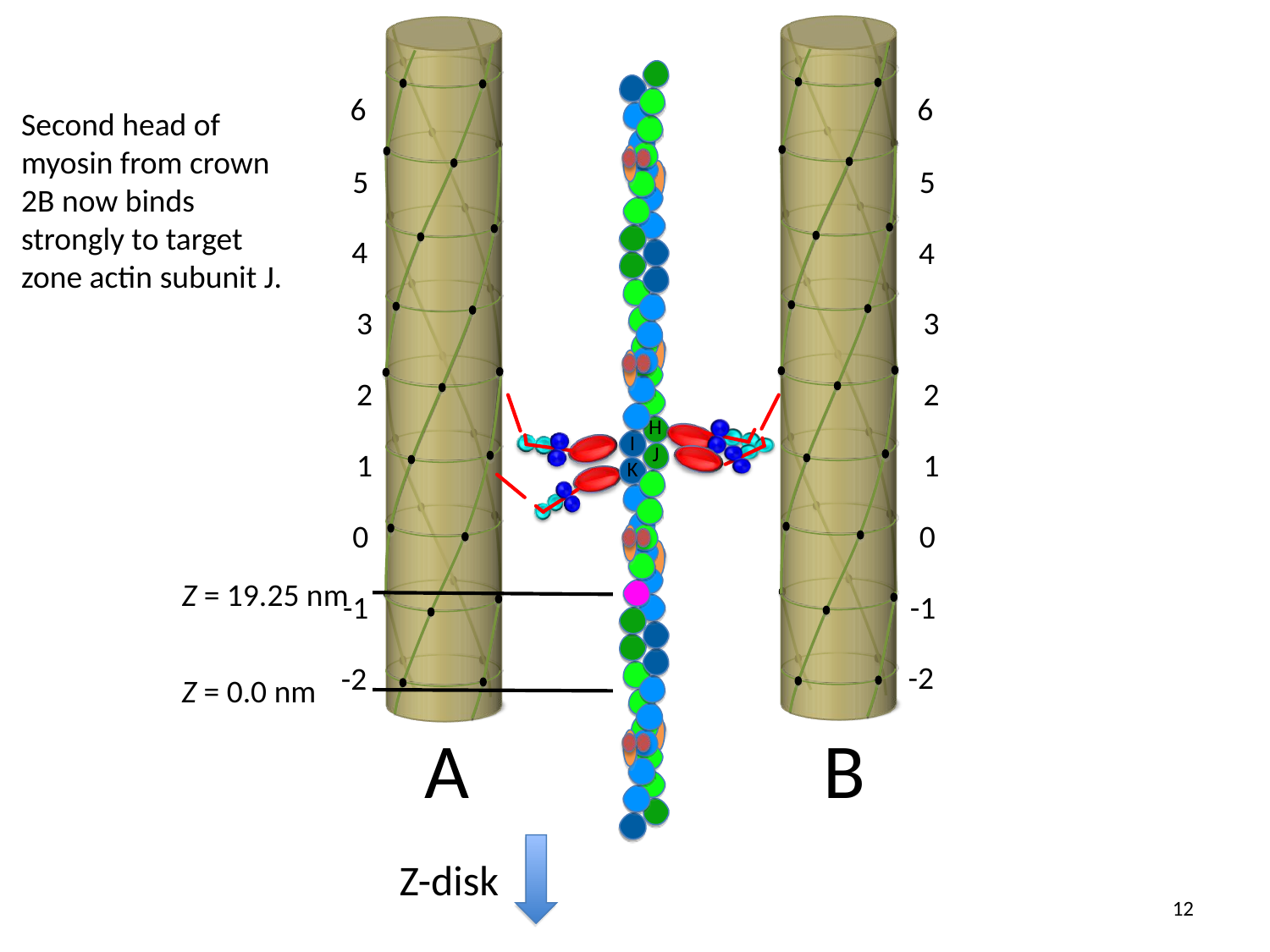

6
5
4
3
2
1
0
-1
-2
6
5
4
3
2
1
0
-1
-2
H
I
J
K
Second head of myosin from crown 2B now binds strongly to target zone actin subunit J.
Z = 19.25 nm
Z = 0.0 nm
A
B
Z-disk
<number>

## Slide 13
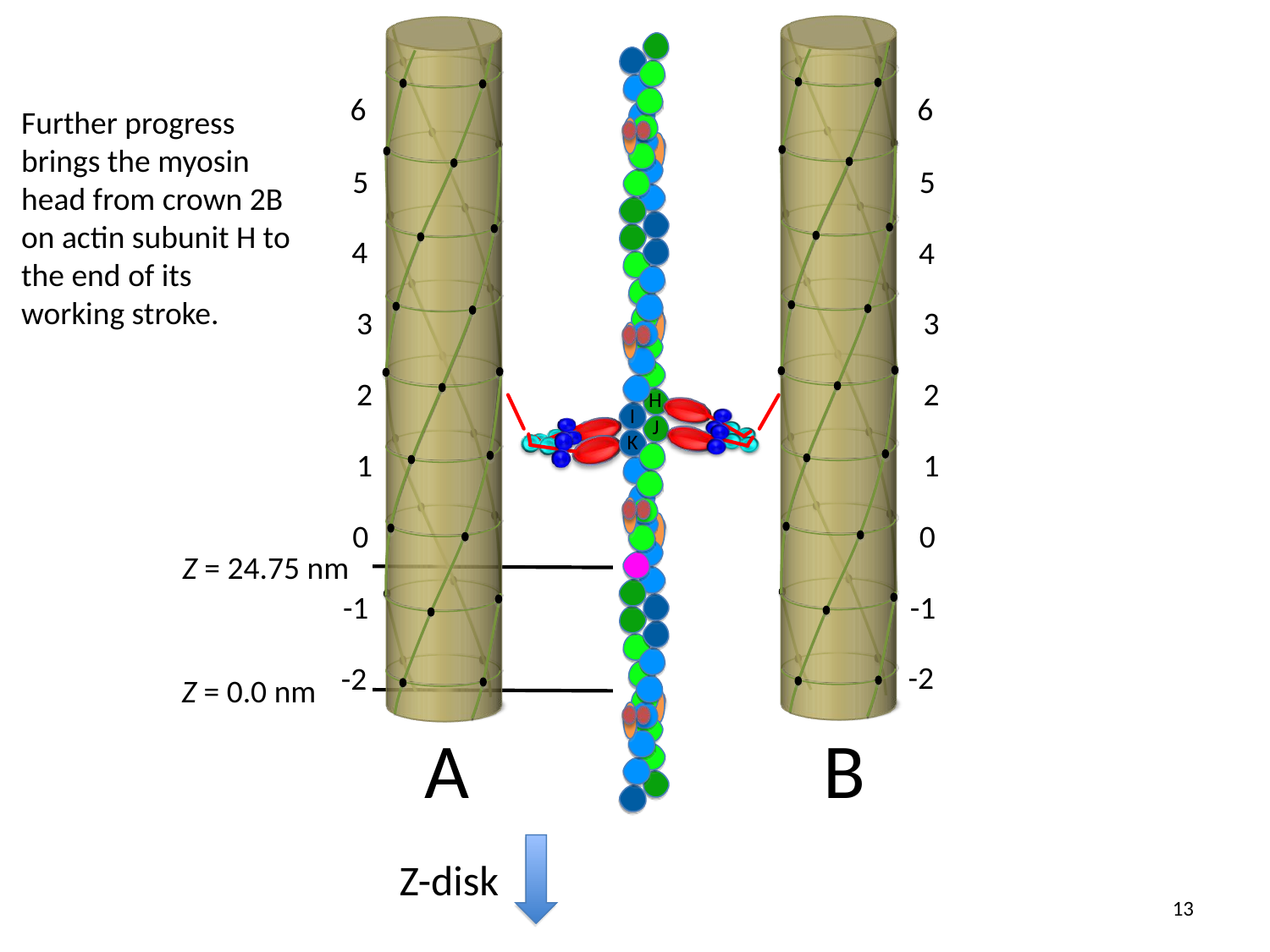

6
5
4
3
2
1
0
-1
-2
6
5
4
3
2
1
0
-1
-2
H
I
J
K
Further progress brings the myosin head from crown 2B on actin subunit H to the end of its working stroke.
Z = 24.75 nm
Z = 0.0 nm
A
B
Z-disk
<number>

## Slide 14
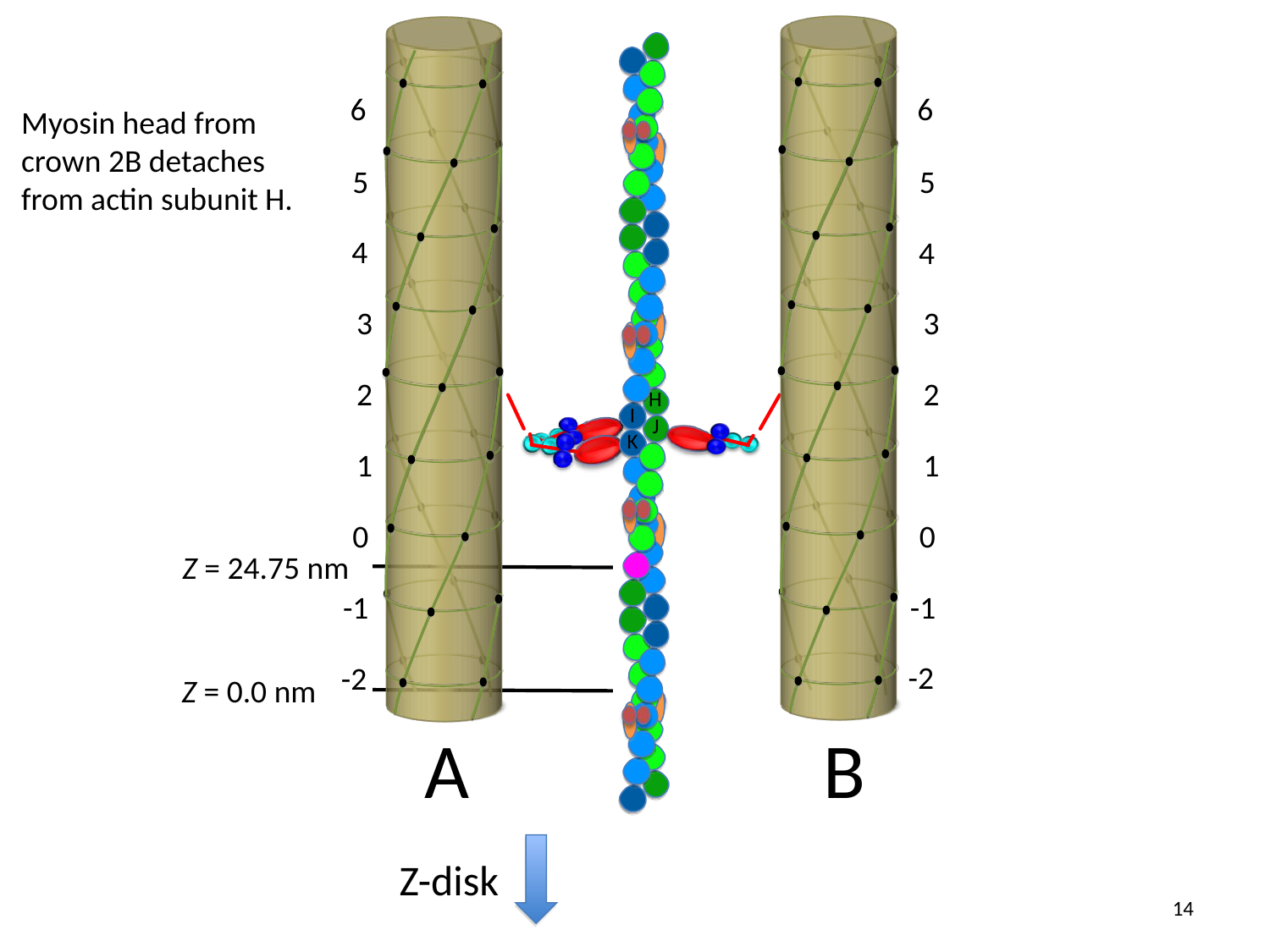

6
5
4
3
2
1
0
-1
-2
6
5
4
3
2
1
0
-1
-2
H
I
J
K
Myosin head from crown 2B detaches from actin subunit H.
Z = 24.75 nm
Z = 0.0 nm
A
B
Z-disk
<number>

## Slide 15
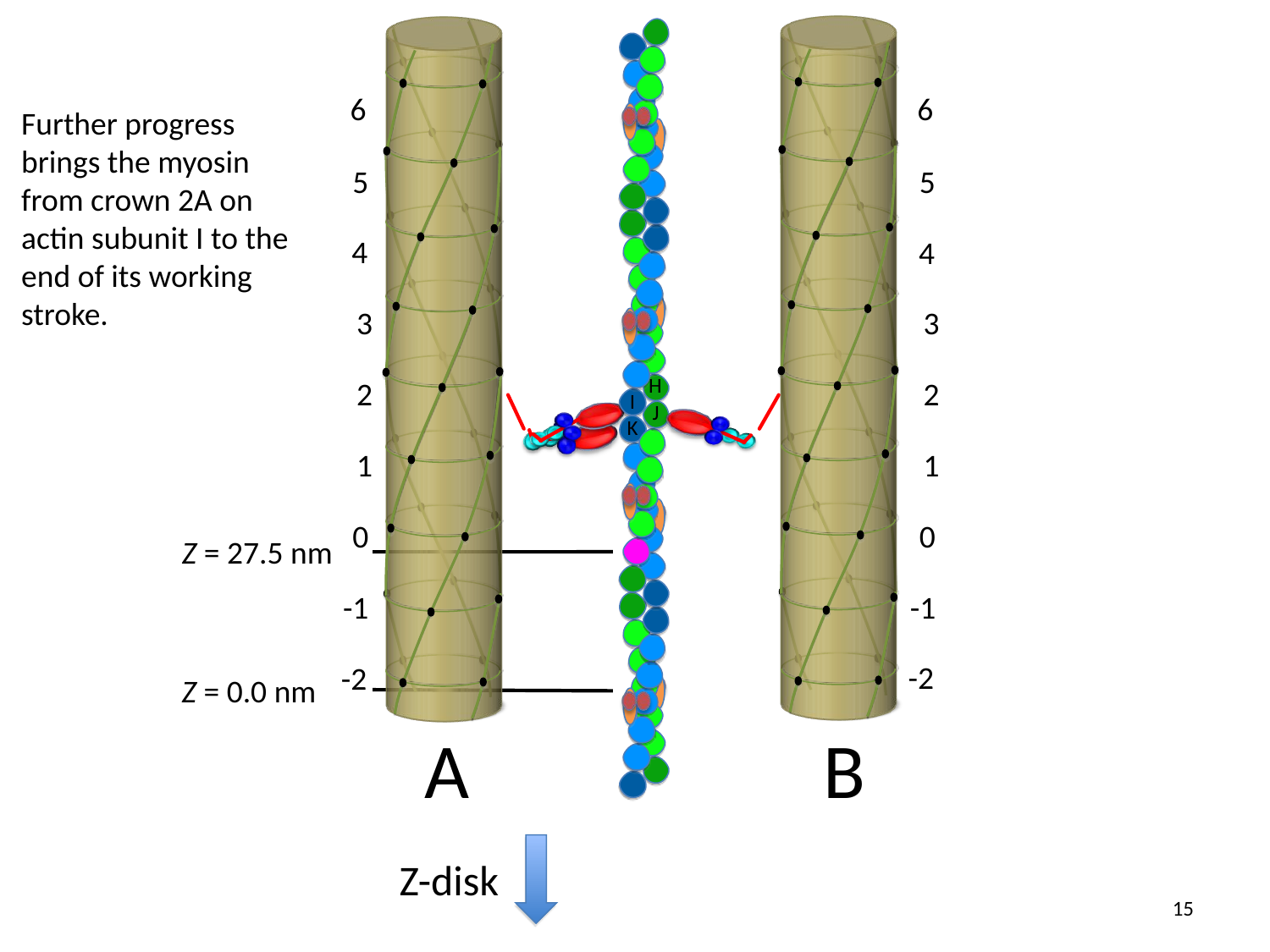

6
5
4
3
2
1
0
-1
-2
6
5
4
3
2
1
0
-1
-2
H
I
J
K
Further progress brings the myosin from crown 2A on actin subunit I to the end of its working stroke.
Z = 27.5 nm
Z = 0.0 nm
A
B
Z-disk
<number>

## Slide 16
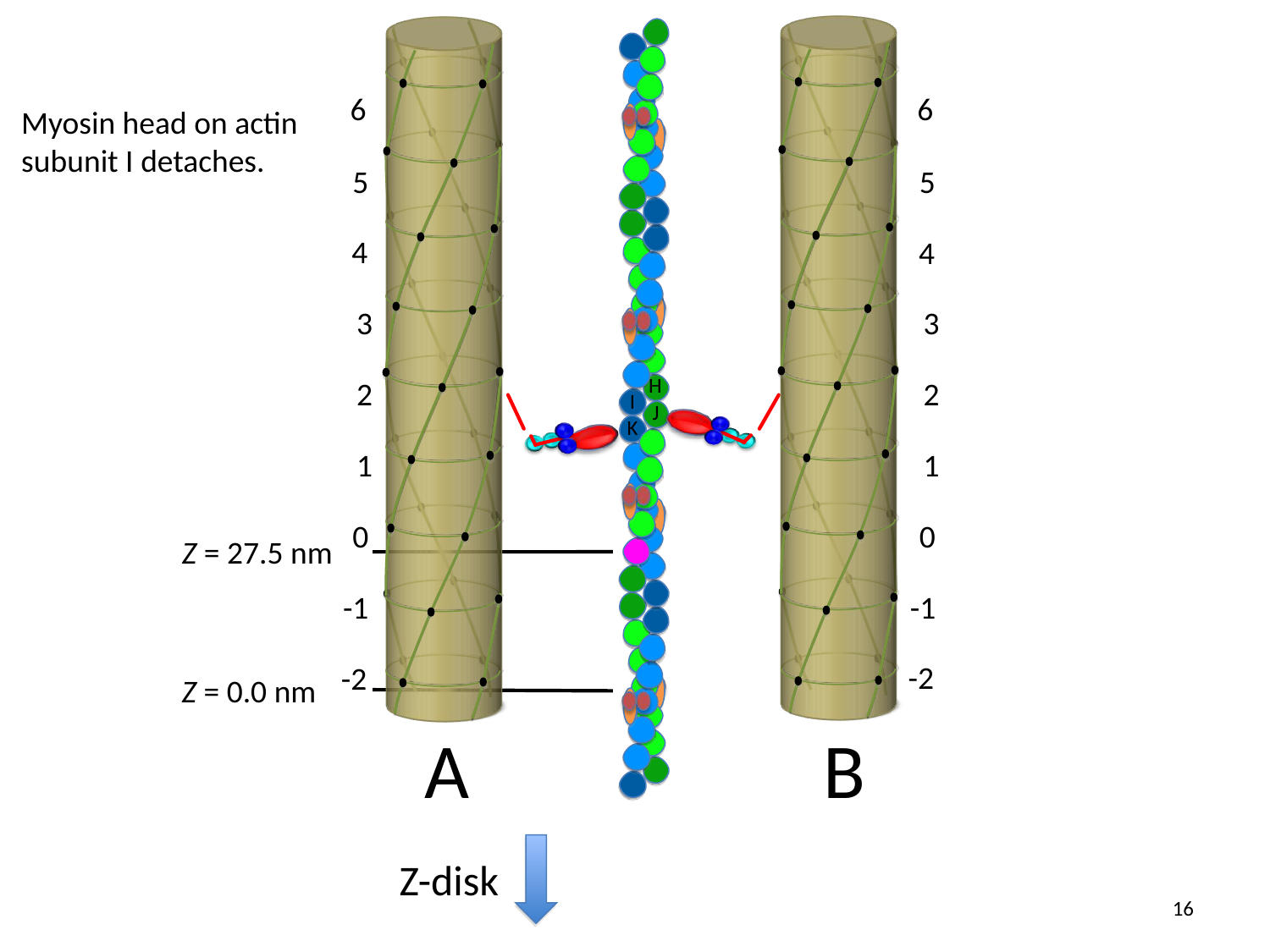

6
5
4
3
2
1
0
-1
-2
6
5
4
3
2
1
0
-1
-2
H
I
J
K
Myosin head on actin subunit I detaches.
Z = 27.5 nm
Z = 0.0 nm
A
B
Z-disk
<number>

## Slide 17
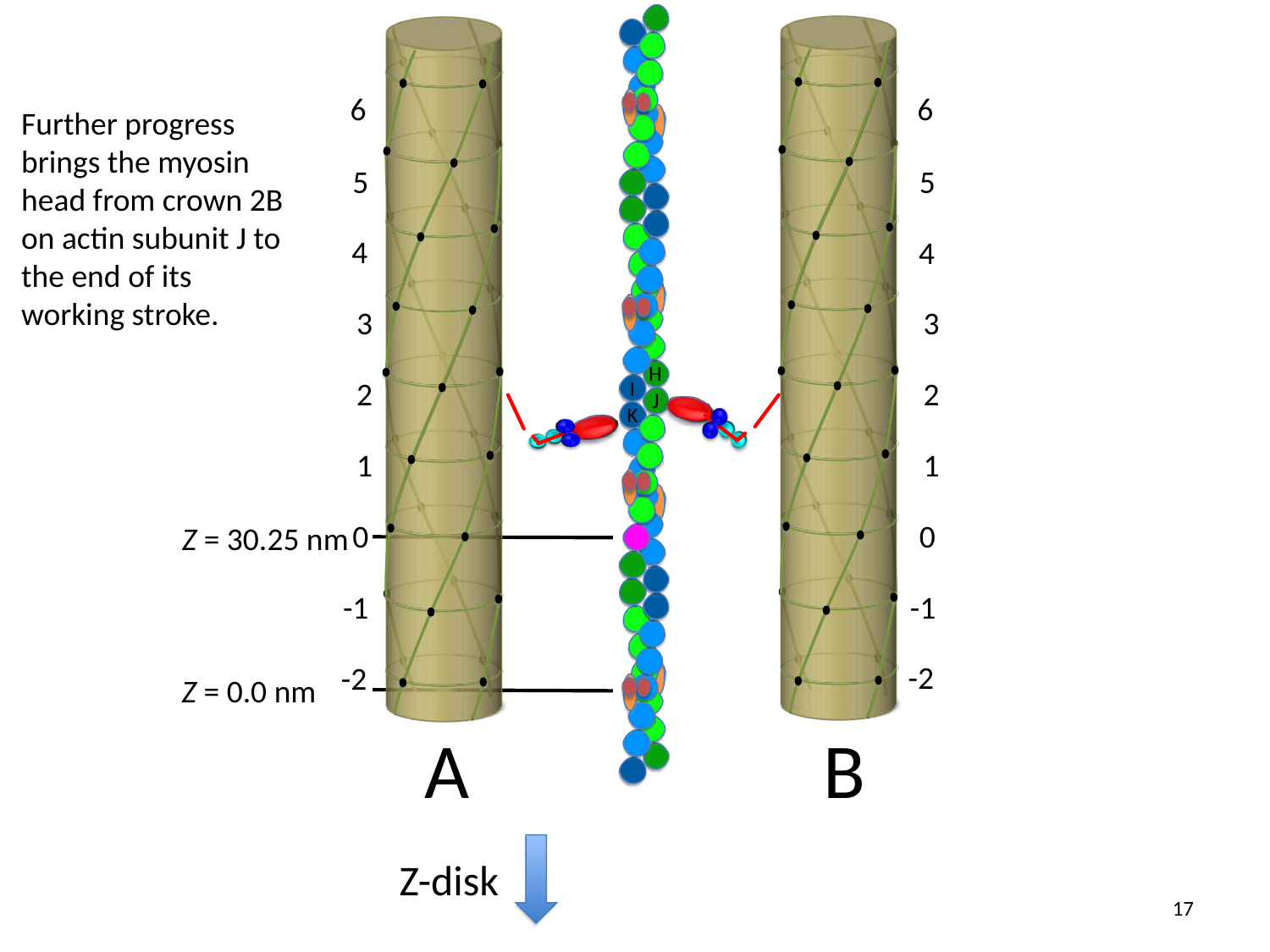

H
I
J
K
6
5
4
3
2
1
0
-1
-2
6
5
4
3
2
1
0
-1
-2
Further progress brings the myosin head from crown 2B on actin subunit J to the end of its working stroke.
Z = 30.25 nm
Z = 0.0 nm
A
B
Z-disk
<number>

## Slide 18
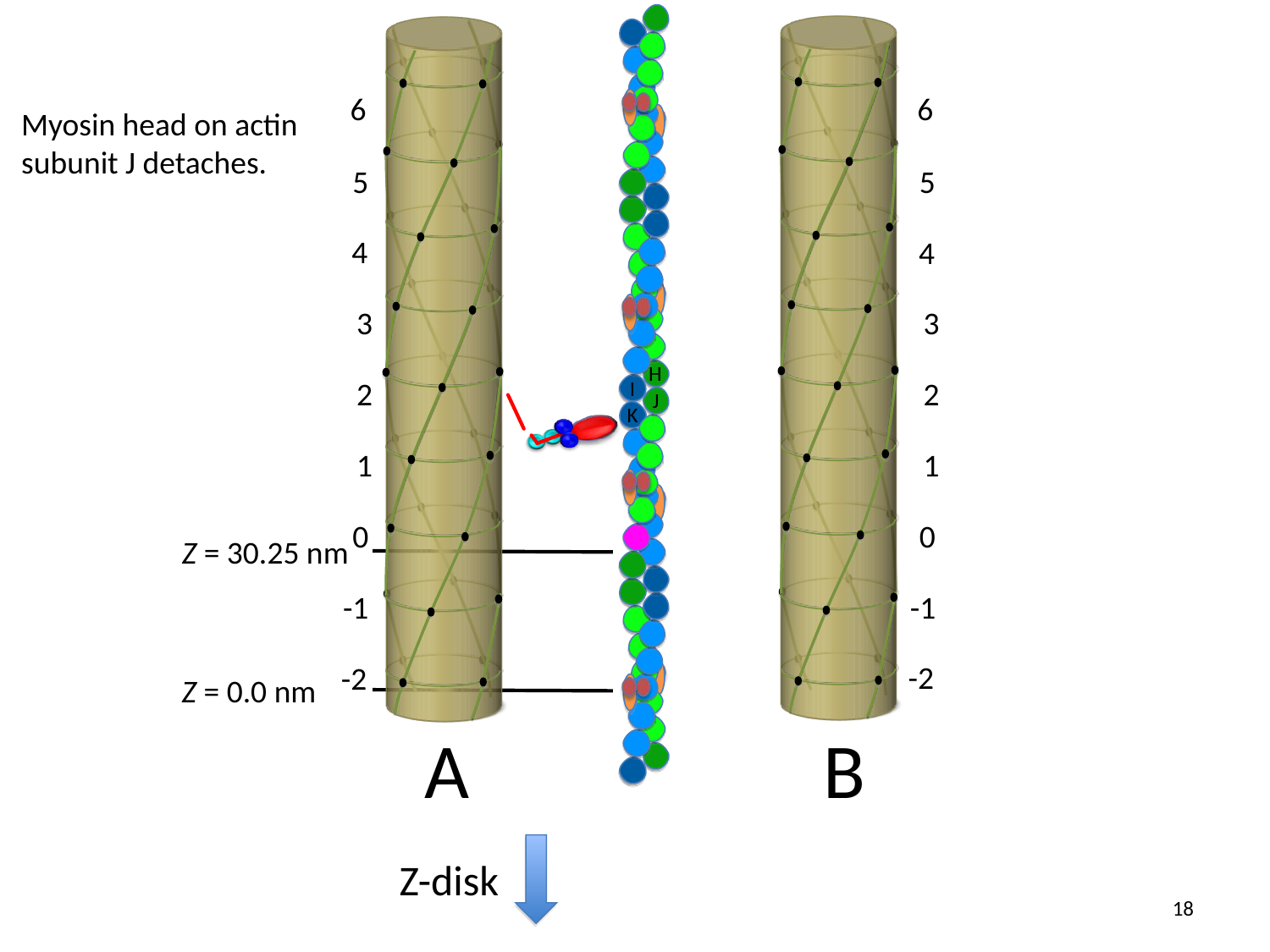

H
I
J
K
6
5
4
3
2
1
0
-1
-2
6
5
4
3
2
1
0
-1
-2
Myosin head on actin subunit J detaches.
Z = 30.25 nm
Z = 0.0 nm
A
B
Z-disk
<number>

## Slide 19
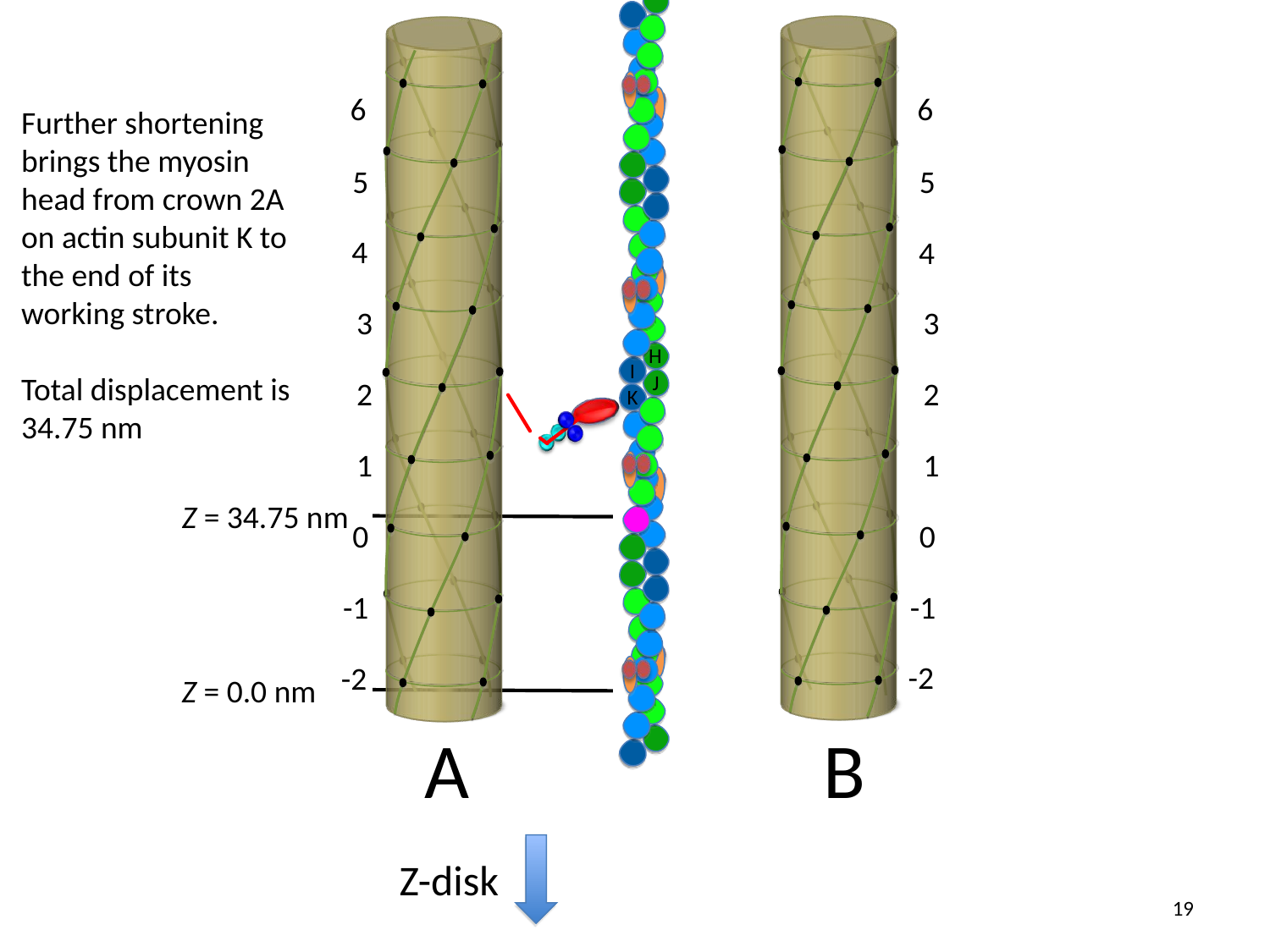

H
I
J
K
6
5
4
3
2
1
0
-1
-2
6
5
4
3
2
1
0
-1
-2
Further shortening brings the myosin head from crown 2A on actin subunit K to the end of its working stroke.
Total displacement is 34.75 nm
Z = 34.75 nm
Z = 0.0 nm
A
B
Z-disk
<number>

## Slide 20
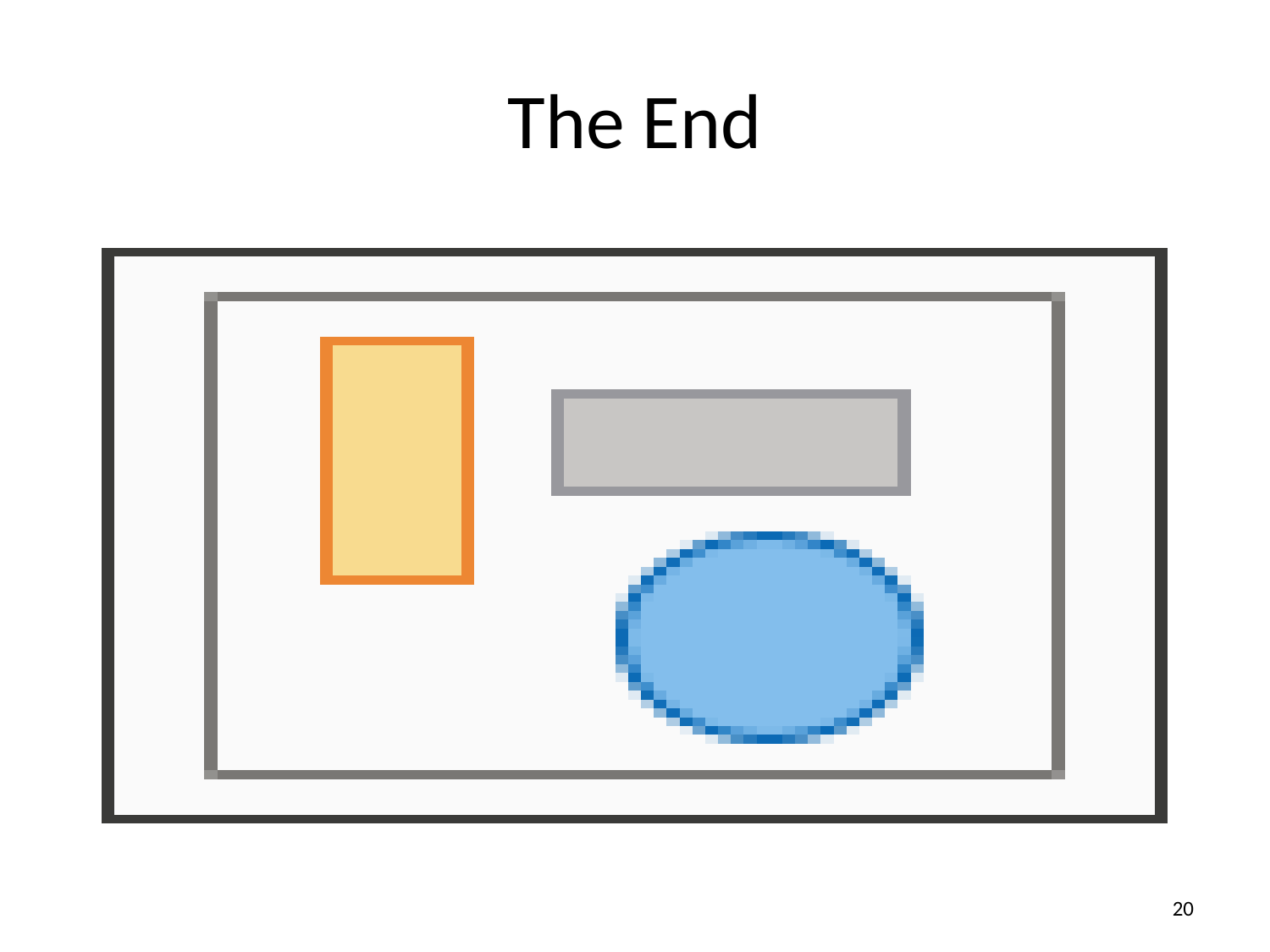

# The End
<number>
